# Insights into population behavior during the COVID-19 pandemic from cell phone mobility data and manifold learning

**DOI:** 10.1038/s43588-021-00125-9

**Published:** 2021-09-22

**Authors:** Roman Levin, Dennis L. Chao, Edward A. Wenger, Joshua L. Proctor

**Affiliations:** 1 Department of Applied Mathematics, University of Washington, Seattle, WA USA; 2 Institute for Disease Modeling, Bill and Melinda Gates Foundation, Seattle, WA USA

**Keywords:** Computational science, Data mining, SARS-CoV-2, Infectious diseases

## Abstract

Understanding the complex interplay between human behavior, disease transmission and non-pharmaceutical interventions during the COVID-19 pandemic could provide valuable insights with which to focus future public health efforts. Cell phone mobility data offer a modern measurement instrument to investigate human mobility and behavior at an unprecedented scale. We investigate aggregated and anonymized mobility data, which measure how populations at the census-block-group geographic scale stayed at home in California, Georgia, Texas and Washington from the beginning of the pandemic. Using manifold learning techniques, we show that a low-dimensional embedding enables the identification of patterns of mobility behavior that align with stay-at-home orders, correlate with socioeconomic factors, cluster geographically, reveal subpopulations that probably migrated out of urban areas and, importantly, link to COVID-19 case counts. The analysis and approach provide local epidemiologists a framework for interpreting mobility data and behavior to inform policy makers’ decision-making aimed at curbing the spread of COVID-19.

## Abstract (web summary)

Combining human mobility data and nonlinear mathematical analysis techniques, this study offers insights into the interplay between human behavior, disease transmission and non-pharmaceutical interventions during the COVID-19 pandemic.

## Main

The ongoing COVID-19 pandemic has had a devastating impact on mortality^1^, morbidity^2^ and economic activity^3^, leading to increased food insecurity, poverty and socioeconomic inequity^2,4,5^. During the first year of the pandemic, most public health interventions attempting to arrest or mitigate the spread of the disease caused by the severe acute respiratory syndrome coronavirus 2 (SARS-CoV-2) were non-pharmaceutical interventions aimed at decreasing transmission by changing people’s behavior. For example, every state in the United States issued mandatory or advisory stay-at-home orders between March and May of 2020^6^. However, characterizing changes in behavior during the COVID-19 pandemic, whether due to adherence to stay-at-home orders, loss of employment or non-pandemic-related factors, is challenging. In this Article, we use smartphone mobility data aggregated by SafeGraph^7^ to identify the heterogeneous mobility behaviors from March to July of the pandemic in four states and reveal consistent motifs across states, within a state and even within urban centers. We compare these behaviors to the numbers of COVID-19 cases in subsequent months. We believe the approach and insights in the work could be leveraged by epidemiologists and integrated with other surveillance indicators to provide public health officials a holistic recommendation as they decide on interventions such as educational campaigns by geographic area and socioeconomic status.

The use of cell phone location data is a relatively new but promising way to quantify human movement. The locations of cell phones can be tracked by service providers or applications installed on smartphones by users, but data that are shared with scientists are typically anonymized and aggregated to protect the privacy of individuals^8–10^. Mobility data offer a unique measurement instrument to link public health statements and related legislative actions taken to reduce population mobility with an actual effect on population behavior. Cell phone mobility data have provided early evidence that these orders were indeed associated with reductions in movement^11–15^. Moreover, adherence was not uniform and may be associated with factors such as socioeconomic status and political leanings^11–15^. The keen interest in cell phone mobility data to help inform policy makers during the COVID-19 pandemic has been widely discussed^16^, with strong emphasis on the challenges facing data ascertainment bias, interpreting the link between mobility and behavior changes, and the lack of a single mathematical framework for analyzing the data^8,9^. So far, most investigations of mobility data during the COVID-19 pandemic have compared summary statistics from mobility data, such as average cell phone mobility within a region, between regions with different demographics. In this Article, we leverage the mobility data at full geographic and temporal resolution along with recently developed mathematical methods from dynamical systems and machine learning to identify patterns of behavior that are consistent across multiple geographic scales and provide insight into behavioral differences.

Analyzing and interpreting high-dimensional mobility time-series is a challenge. Model and dimensionality reduction has a rich history in the analysis of dynamical systems, with early theoretical work on bifurcation analyses enabling the categorization of qualitatively different dynamic regimes^17^ to the more recent data-driven, equation-free approaches^18^ enabled by advances in machine learning and pattern analysis^19^. The standard approach typically involves a linear dimensionality reduction technique, such as singular value decomposition (SVD)^20^, in conjunction with a statistical clustering model to identify similarities across time-series^21^. Despite the broad success of this approach, substantial limitations have been identified due to the underlying assumptions associated with SVD and the mismatch with characteristics of data collected from a complex, temporally evolving system. This discrepancy has motivated the development of a diverse set of nonlinear dimensionality reduction techniques for time-series data^22^. Methods such as diffusion maps and Laplacian eigenmaps, popular in statistical and computational analyses^23,24^ and contained in a class of machine learning methodologies called ‘manifold learning’, have been utilized by the dynamical systems community to identify nonlinear embeddings of the dynamics directly from observational data from the system^22^. Success has been demonstrated with these methods using data generated from simulation models^25^. Here, we leverage these methodologies to identify a lower-dimensional embedding of the mobility time-series data, providing a framework that highlights common mobility behaviors at the census-block-group (CBG) scale, identifies the geographic connectivity of behavior at different spatial scales, and reveals insights into epidemiologically relevant subpopulations.

## Results

### Identifying patterns in CBG stay-at-home behavior

**Fig. 1:**
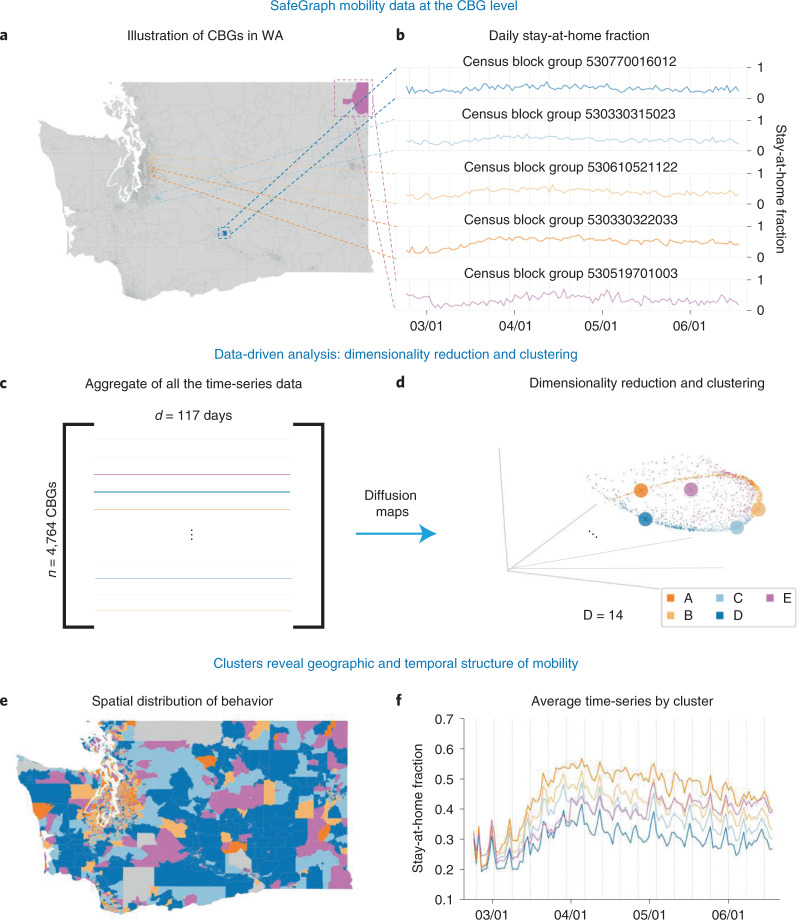
Overview of the analysis workflow for mobility data. **a**, CBG borders shown on the map of Washington state. Five example CBGs are highlighted by five different colors on the map. **b**, The stay-at-home time-series from SafeGraph data are plotted for the five CBGs (the CBG Federal Information Processing codes are shown above each plot). **c**, All CBG SafeGraph time-series are aggregated in a matrix form. **d**, Diffusion maps are applied to the data matrix. All of the CBG time-series are represented as points in the first three dimensions of a 14D Laplacian eigenmaps embedding. The colors correspond to a five-cluster (A, B, C, D and E) GMM model applied to the data in the 14D embedding space. The larger dots represent the same example CBGs from **a** and **b**. **e**, Each GMM cluster of CBGs is plotted on the Washington map, with each color representing a different cluster. **f**, The average mobility time-series plotted for each cluster. Source data

**Fig. 2:**
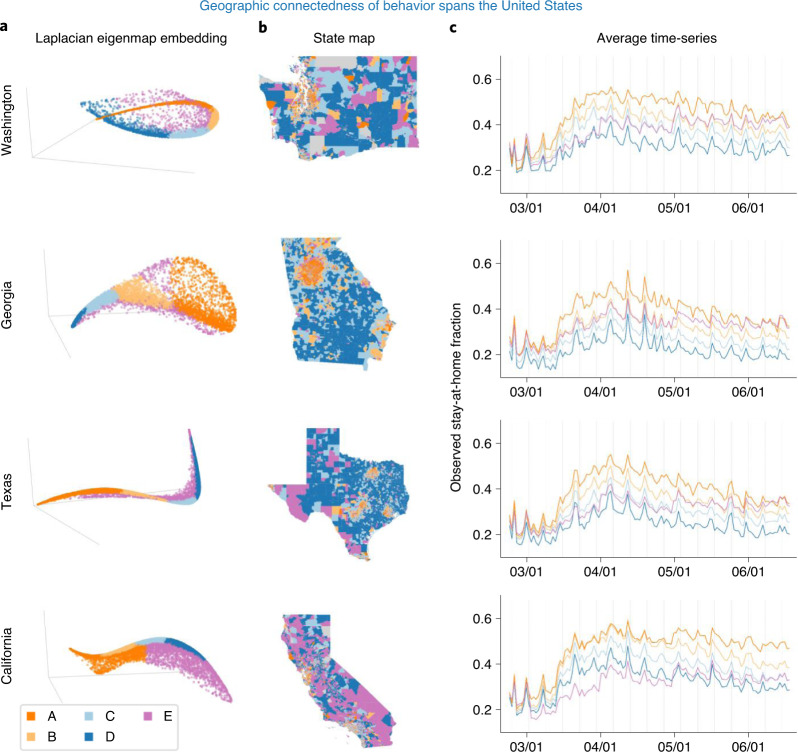
Clustering results are consistent across four states. **a**, 3D Laplacian eigenmap visualization of the data manifold for each state. **b**, Mobility clusters (A, B, C, D, E), by color, for each state. **c**, Average mobility time-series for each cluster. Clusters are highlighted by color. Clustering was done in a 14D (optimal) embedding space. Source data

The SafeGraph stay-at-home data offer insights into the levels and trends of human mobility at the CBG geographic scale during the 2020 COVID-19 pandemic in the United States (Fig. 1). Nonlinear dimensionality reduction of the time-series data from Washington state revealed a low-dimensional embedding providing insight into the consistency of mobility behavior across CBGs (Fig. 1d). Moreover, the embedding and stay-at-home behaviors for Washington are qualitatively similar to those of Georgia, Texas and California (Fig. 2). The optimal embedding dimension was 14 for all four states, determined by the trustworthiness metric (section ‘Manifold learning and nonlinear dimensionality reduction’ and Supplementary Section 2). A similar low-dimensional structure in the time-series data can be found with a diversity of nonlinear dimensionality reduction methods, including diffusion maps with fixed- and variable-bandwidth kernels that have different underlying assumptions about the data distribution^22,26,27^ (Supplementary Section 2). By contrast, clustering in the linearly reduced space is highly uncertain (Supplementary Section 1).

The low-dimensional embedding provides insight into the similarity of stay-at-home behavior between CBGs. Figure 2 provides a visualization in three embedding dimensions of this coherent structure. Note that, for each state, certain CBG time-series are more similar to each other and the visualization indicates a large density of CBGs along a distinct, tubular data manifold. Fitting a Gaussian mixture model (GMM) to the stay-at-home time-series in the 14-dimensional (14D) embedding space identifies four clusters for California, Texas and Washington and five clusters for Georgia (based on knee-point detection in the Bayesian information criterion (BIC) described in section ‘GMM clustering and uncertainty quantification’). For subsequent analyses, we chose five as the number of clusters for every state due to the optimal clustering for Georgia, comparisons across the four states (Supplementary Table 3) and the geometric structure of the data (described in the next section). Figure 2 illustrates how the clustering model groups CBGs in the embedding space (Fig. 2a), and the average mobility time-series for each cluster (Fig. 2b) highlight the difference in stay-at-home behavior by cluster within a state and also the consistency across all four states. The cluster assignments were robust to model initialization (Supplementary Section 3) and had low associated uncertainty values (Supplementary Section 2). The number of clusters and cluster assignments were optimized according to a standard approach that balances model fit and parsimony (section ‘GMM clustering and uncertainty quantification’), but the number of clusters could be changed depending on a desired level of granularity, or a continuous colormap could be used (Supplementary Section 4).

One clear difference between the clusters is their average level of mobility. For example, in Washington, the average staying-at-home level increases from the CBGs in the dark blue cluster (cluster D) to the bright orange cluster (cluster A) (Fig. 1f; representative CBG time-series for each cluster are shown in Figs. 1b and 2). The order of the clusters along the dense data manifold in the embedding space is aligned with their mean staying-at-home fraction (Fig. 2). The average time-series for clusters D through A do not intersect and are aligned in increasing order on the *y* axis. However, the purple cluster, E, does not follow a similar trend with respect to the dense data manifold, nor the average time-series. For this cluster, we find that the fraction of the devices staying at home increases sharply in May 2020. A similar motif consistently occurs across each state (Fig. 2); cluster E primarily captures outliers from the primary bulk trends that are continuously distributed across clusters A, B, C and D. Those outliers are linked to a variety of important subpopulations, explored in more detail in the section ‘Identifying areas with likely population turnover’. We also find that the CBG clustering and average time-series by cluster also indicate that the change of behavior over time is different across clusters before April (Supplementary Section 6).

### Geographic relationship of CBGs and mobility patterns

The CBGs within each mobility cluster (defined in the previous section) are geographically connected and have consistent patterns across all four states. The second column of Fig. 2 illustrates these broad trends, which are most visually evident in the distinction between urban, peri-urban and rural areas. We found that counties associated with large metropolitan areas had relatively high proportions of clusters A and B, while rural counties had the highest proportions of cluster D (Supplementary Fig. 16). For example, in Washington, the Seattle-area CBGs mostly belong to the bright orange and light orange most-staying-at-home clusters A and B, and the same is true for nearby Bellevue and Redmond. Similarly, in Texas, three large orange regions correspond to Dallas, Houston and Austin. In Georgia, the distinct orange area on the map corresponds to Atlanta, and in California we see orange colors around San Francisco, San Jose and Los Angeles areas. Similarly, blue colors—clusters C and D with lower stay-at-home levels—form continuous regions in rural areas on the state maps. CBGs that are close geographically tend to have similar mobility patterns.

**Fig. 3:**
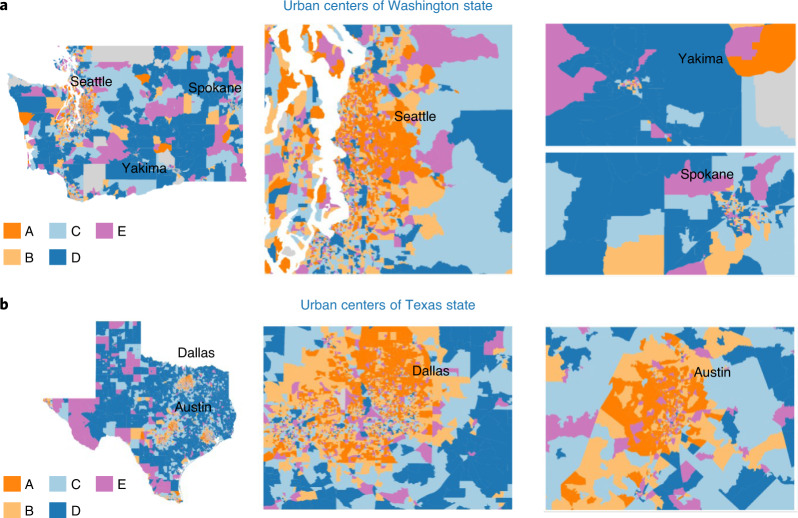
Mobility clusters in metropolitan areas. **a**, Clusters in Washington state and selected urban areas. **b**, Clusters in the state of Texas and selected urban areas. Source data

Within each state, there is a stark contrast between urban and rural areas (Fig. 2). For example, in Washington, the large metropolitan areas around Seattle and Bellevue are colored orange (clusters A and B), as opposed to larger rural CBGs, which belong to blue clusters (C and D). Large cities like Spokane or Yakima also have dense orange coloring (Fig. 3), suggesting that changes in behavior within urban centers are similar, despite being geographically quite distant from each other. The time-series in Fig. 2c show that urban areas (orange clusters A and B) stay at home substantially more than rural areas (blue clusters C and D). This observation is consistent across all four states.

This analysis also identifies heterogeneity within the geographic scale of urban centers and rural areas. For example, in Dallas and Seattle there are urban CBGs that belong to blue clusters C and D, indicating that they stay at home less than the surrounding areas (Fig. 3). Moreover, populous cities such as Seattle, Atlanta, Austin and Dallas have distinct geographic groupings of CBGs for clusters A and B within the urban area (Fig. 3; Supplementary Section 5 describes clustering in Atlanta). Figure 2a clearly presents a smooth transition in the Laplacian eigenmap embedding space between the bright orange cluster A that stays at home the most to the dark blue cluster D that stays at home the least. Remarkably, we observe the same on the geographic map. For example, there is a rough radial pattern around Dallas and Austin: bright orange CBGs densely cover the city center and are replaced by light orange, then light blue and eventually dark blue as the distance from the city center increases (Fig. 3). That is, the transition is quite consistent—it covers the intermediate colors and the stay-at-home level gradually decreases as distance from the city increases, suggesting a more nuanced interpretation about the continuity of behavior across CBGs within urban centers supported by the geometric structure of the data manifold. In the greater Seattle area, the transition is substantially less pronounced, especially moving eastward from downtown. Note that both a large urban area (Bellevue) and suburb (Redmond, the home to Microsoft) exist to the east of Seattle, both with a higher-income population.

Despite the optimal number of clusters being four or five for each state, relaxing this criterion and allowing for more clusters provides more granular information within and around urban areas while maintaining consistency with the optimal GMM. This also follows the intuition provided by the illustrations of the nonlinear embedding in three dimensions (Fig. 2a); namely, the embedding is broken into finer-grained clusters, enabling higher-resolution comparisons between CBGs. Supplementary Section 4 provides details on altering the number of clusters. Furthermore, a continuous mapping of the data along the dense tubular structure of the data manifold shows the smooth transition across urban, peri-urban, suburban and rural areas (Supplementary Section 4). By contrast, purple cluster E is not wholly on the tubular structure and does not exhibit the same geographically connected characteristics as the other clusters. More detail is provided on this cluster and the possible difference in its subpopulation structure in the section ‘Identifying areas with likely population turnover’.

### Linking income, population density and behavioral data

**Extended Data Fig. 1:**
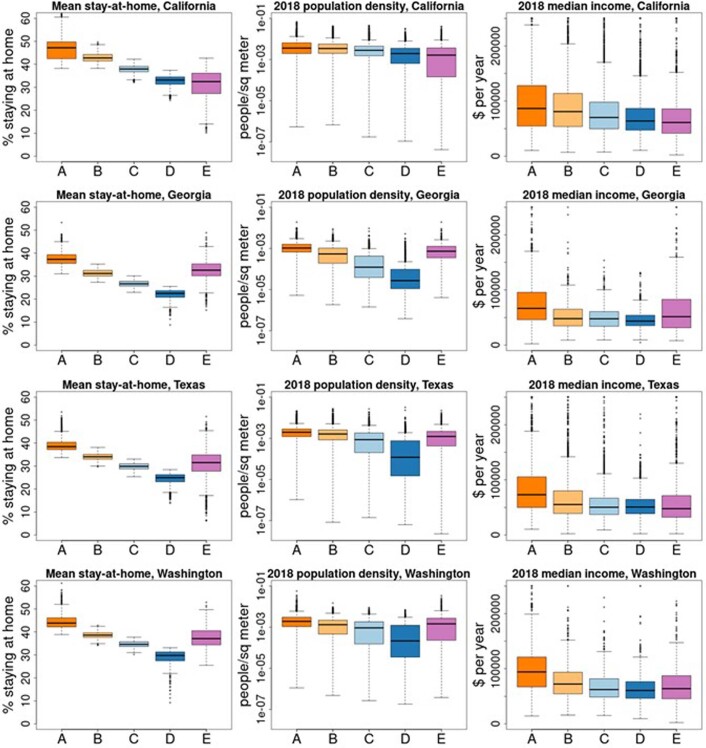
Distribution of stay-at-home behavior and demographic covariates by cluster. The boxplots present the interquartile range (boxes) and median values (center horizontal lines) of the covariate values for CBGs in each of the five clusters. Whiskers span the 95% range. Points represent outliers outside of the 95% range. The total number of points in each mobility cluster can be found in Supplementary Table 3. The ‘mean stay at home’ fraction of a CBG is the mean of the daily percent of mobile devices that stayed completely at home during the time period analyzed.

Clusters A and B, which, on average, stayed home the most, included the most densely populated CBGs, while clusters C and D included the more sparsely populated ones (Extended Data Fig. 1). High population density is generally an indication of urban populations and low density an indication of rural areas (see the maps in Fig. 2). The CBGs in clusters A and B also had the highest median household incomes (Extended Data Fig. 1). In all states, the median stay-at-home fraction, population density and household income of CBGs had a consistently decreasing trend from clusters A to D, and the Jonckheere–Terpstra test rejects the null hypothesis that these four clusters come from the same distribution of values (*P* < 0.01). Cluster E did not follow these trends and appeared to cover a wider range of values (Extended Data Fig. 1).

**Fig. 4:**
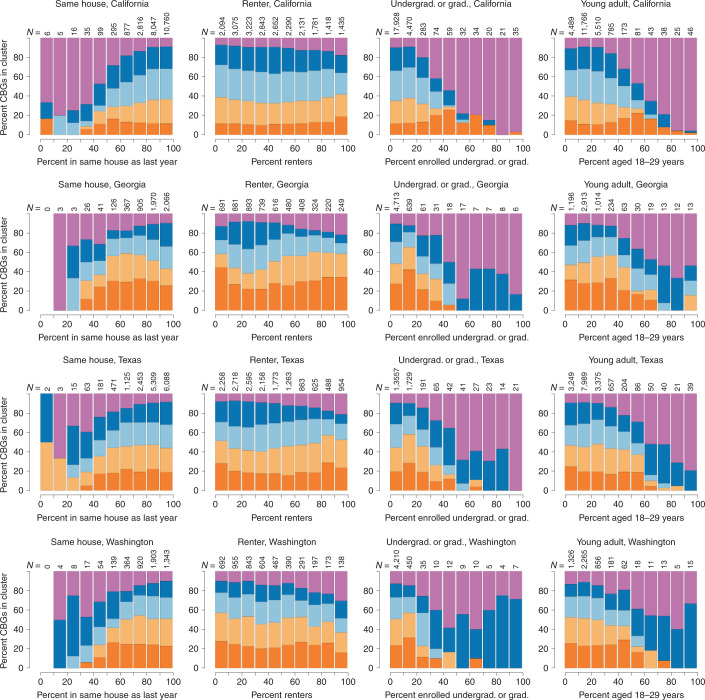
Proportions of CBGs in each cluster by population characteristics associated with geographic mobility. The fraction of a CBG’s population associated with the characteristic is plotted on the *x* axis. CBGs are partitioned into 10 equally spaced bins, defined by the proportion of each CBG’s population having the characteristic in 10% increments. The numbers of CBGs belonging to each bin are printed along the top of each panel. The proportion of CBGs in each cluster is plotted as vertically stacked bars for each bin (with cluster A in dark orange on the bottom through cluster E in purple on the top). Note that some bins contain only a handful of CBGs, so specific proportions should be interpreted with caution. Source data

Cluster E has a higher proportion of people who we expect to have high ‘geographic mobility’ (that is, change residences frequently). Using estimates from the 2018 American Community Survey (ACS), CBGs with a low proportion living in the same house in the previous year or a high proportion of renters, people enrolled in undergraduate or professional degree programs, or who are ‘young adults’ (18 to 29 years old) tended to be in cluster E (Fig. 4). In California, the proportion of people with high geographic mobility appears to be higher in cluster A than in cluster B.

**Extended Data Fig. 2:**
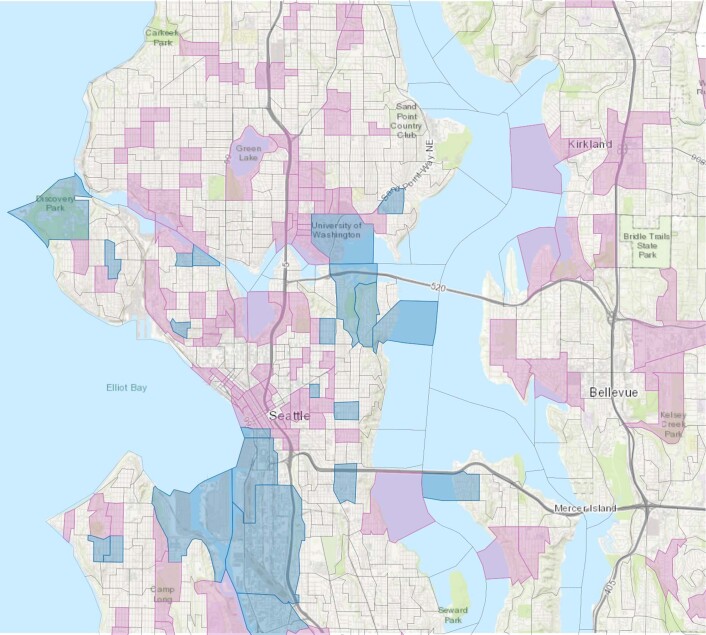
Clusters in the Seattle metropolitan area. Census block group boundaries are outlined. CBGs belonging to clusters D and E are highlighted in dark blue and purple, respectively.

Upon closer investigation of the location of clusters in the city of Seattle, Washington, the spatial distribution of clusters D and E is consistent with the associations described above (Extended Data Fig. 2). The area surrounding the University of Washington, where a large number of undergraduate and graduate students live, is in cluster E, while the university itself is in cluster D (Extended Data Fig. 2, center of map). Cluster E also includes downtown and Lake Union, where a recent influx of young tech workers fueled the development of new apartments. Interestingly, in addition to students and young tech workers in Seattle, cluster E also indicates some very high median income populations on the waterfront of Bellevue and Kirkland that were also highly mobile during this period. Cluster D includes ‘SODO’, the industrial area southwest of downtown, which is less affluent than the populations to the west, east and north.

### Identifying areas with likely population turnover

The available SafeGraph dataset does not allow one to track the movements of individuals, but there are trends consistent with high population turnover. One can track the number of mobile devices that are detected by SafeGraph each day but not in their ‘home’ CBG on a given day, which we call ‘never-near-home’ devices. These devices could be on a trip away from home or they could have moved away entirely.

**Fig. 5:**
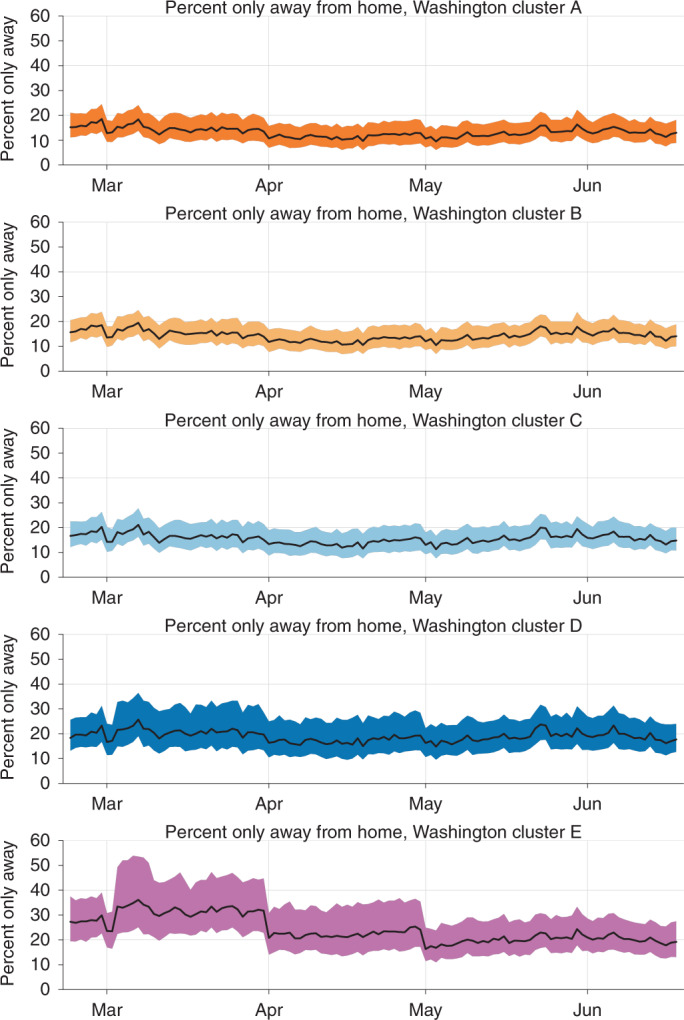
The fraction of devices that are only away from their homes each day. The black curves are medians and the shaded areas are interquartile ranges (the 25th to 75th percentile of ‘percent only away’ observed in CBGs on a given day) for the CBGs in each cluster in Washington state each day. The total number of points in each mobility cluster is provided in Supplementary Table 3.

In March, the fraction of never-near-home devices was highest in cluster E (Fig. 5 and Supplementary Fig. 17). On 1 April and again on 1 May, the number of devices never near home drops sharply in cluster E but not in the other clusters. This behavior is consistent with the owners of these devices moving to a new residence and SafeGraph reassigning these devices to the new residence on the first day of a subsequent month. These home locations were updated by SafeGraph at the start of each month until mid-May, when SafeGraph changed its procedure for assigning home locations to devices^28^. The high proportion who were never near their ‘homes’ in March and April and the sharp drops in these fractions on 1 April and 1 May in cluster E, and to a lesser extent in cluster D, are consistent with this population moving away. In California, cluster A also has a noticeable decline on 1 May (Supplementary Fig. 17), which could indicate a high-income group that is geographically mobile. If a large number of people in a region move away, the devices will appear to be ‘never near home’ because their home locations are out of date. These clusters will appear to be staying at home less than they really are. This batching artifact appears to be resolved in May 2020, and the stay-at-home fraction in cluster E rises relative to the other clusters.

### Linking population mobility patterns to COVID-19 cases

**Fig. 6:**
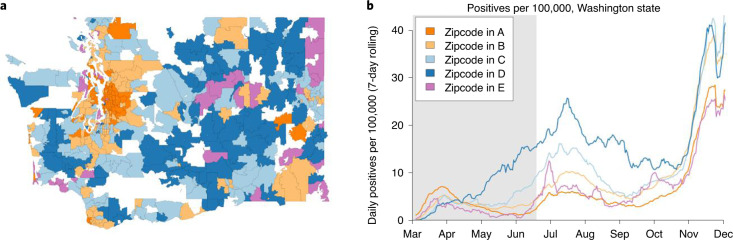
COVID-19 cases by zip code and mobility cluster. **a**, Map of mobility clusters associated with the zip codes of Washington state. The zip codes were assigned to clusters based on the clusters of the CBGs that comprised them. **b**, The daily number of daily COVID-19 cases (that is, SARS-CoV-2 positive tests) per 100,000 population in 2020 smoothed using a seven-day rolling window. The gray region from late February through mid-June indicate the days of mobility data used for the analysis.

The five mobility clusters had distinct epidemic trajectories in Washington state. COVID testing data for Washington state were available at the resolution of zip codes, which are usually but not always larger than CBGs. To map the CBG-level mobility cluster assignments to zip codes, we assigned each CBG to a zip code as described in Methods, and each zip code was assigned the cluster containing the largest share of its population. In general, the zip codes assigned to clusters A and B were near large cities, while rural zip codes were generally in clusters C and D (Fig. 6a). Cluster E appeared in a few urban zip codes (in Seattle and Spokane) and a handful of rural ones. We found that the clusters with the highest mobility from late February through mid-June, clusters C and D, later had the highest number of cases per capita in Washington state. Clusters A and B, which stayed home the most in the first months of the pandemic, had the fewest cases per capita in the summer and fall waves (Fig. 6b). Cluster E, which includes neighborhoods adjacent to the University of Washington’s main campus, had spikes in cases in late June and September, unlike the other clusters. We should note that we used static estimates from the 2018 ACS to compute the per capita number of COVID cases, which might not be appropriate for cluster E, which had a high proportion of enrolled college students (Fig. 4) and a high proportion who may have moved away early in the pandemic (Fig. 5).

## Discussion

Our results are consistent with other studies linking demographic characteristics to cell phone mobility data during the 2020 SARS-CoV-2 pandemic. Two recent studies using data from SafeGraph found that mobile devices from areas with higher median household incomes stayed home more than devices from lower-income areas^13,15^, and this trend occurs in other mobile device datasets^11^. These studies hypothesize that the relationship between income and mobility is due, in part, to the ability of people with high-paying jobs to work from home. A survey found that about half of adults in Seattle switched to telework because of COVID, with high-income households making the change far more than lower-income households (79.3% in households making more than US$150,000 per year and 23.5% among those making less than US$50,000)^29^. A recent study using another source of cell phone mobility data found that mobility was reduced more in urban than rural England^14^, indicating that these trends could generalize beyond the United States.

Several related studies cluster mobility time-series by a single demographic characteristic selected a priori, such as income^11,13,15^, population density^14^ or party affiliation^12^, to demonstrate behavioral differences with respect to that characteristic. Alternately, one could reduce the time-series to a summary statistic, such as average proportion of smartphones that stay at home over a particular time window, and study the relationship between that metric and several demographic covariates. If we had clustered CBGs by average behavior over time, we would still have found that the number of cases was highest among those who stayed home the least, but we would have completely missed the population that migrated early in the pandemic and had distinct outbreaks (Supplementary Fig. 19). Identifying the population that moved early in the pandemic is a direct consequence of using a data-driven, equation-free approach.

The clusters of CBGs that stayed home the most during the first few months of the pandemic had the lowest number of cases per capita later in the following summer and fall waves. Broadly, the research community has found defining a consistent temporal relationship between mobility indicators and transmission challenging. For example, early in the pandemic, researchers found an association between mobility indicators and COVID-19 cases^30,31^, but this did not hold when including data from later in the pandemic^32,33^. In our approach, we identify populations with differences in mobility early in the pandemic that suggest differences in behavior that persist throughout the pandemic even in the face of changing restrictions and behavioral trends. It is likely, though, that mobility contributes to, but does not fully capture, SARS-CoV-2 risk. Mobility may be a proxy for potential exposure to outside the household, but also reflects demographic and socioeconomic factors that affect the persistence of risk for SARS-CoV-2 susceptibility and transmission. Supplementary Section 8 provides additional discussion on linking mobility to SARS-CoV-2 transmission.

We acknowledge several limitations of the mobility data and challenges in linking behavior to demographic variables. SafeGraph aggregates mobility data from many independent sources on the locations of millions of smartphones. These data are obtained from third parties that collect smartphone location and limited information about the devices and their users^10^. Users could opt out of location tracking, but this might not be practical when using smartphone apps that require this information. Children are particularly vulnerable and potentially unable to make informed decisions about tracking, so US federal law restricts online services from collecting information on children under 13 years old^34^. Therefore, conclusions drawn from mobility data are limited to older children and adults. The data used in this study are aggregated by CBG and filtered to preserve the privacy of the mobile device owners. It is difficult to ascertain how well a set of mobility data represents the general population^8,9^. It may also be hard to correct for the fact that different states and segments of the population may have different levels of coverage, including over the course of the pandemic^35^. This is further complicated by likely gaps in coverage for high-risk populations such as migrant agricultural workers. The sourcing of location data from an undisclosed and evolving set of third parties could introduce biases that would be difficult to detect. However, the trends of similar mobility metrics from the different sources—SafeGraph, PlaceIQ and Google data—are qualitatively similar^11^ (Supplementary Section 7 provides more detail). In addition, the associations we found between mobility and other factors are consistent with those found in other datasets and are quite plausible^11,13^.

In future work, we anticipate leveraging this framework to integrate similar data sources, such as Facebook and Google’s mobility data, across a much wider geographic scope to minimize the bias of any one source. We studied the fraction of mobile devices that stayed at home each day, but this is just one metric than can be derived from the mobility data. Other measures, such as the mean length of time spent outside the home, the distance traveled from the home, or even the number of trips to stores, could provide additional insight into the population’s response to the pandemic. The demographic data in this study was from the 2018 American Community Survey, which we believe generally reflects the population in 2020 but might not accurately characterize the demographics of the most rapidly changing areas. We cannot establish the direct cause of the differential reductions in mobility using these data. We use demographic and socioeconomic variables at the census block group level, which could lead us to ecological fallacies, and many of these variables are tightly linked, thus, disentangling their effects is not straightforward and could be counterproductive.

Despite these challenges, population mobility data and connections to behavior can complement other surveillance data to inform public health policy makers. Population behavior is a key component to understanding disease transmission dynamics, and mobility data and the methods contained in this Article help quantify population behavior and the associated COVID-19 epidemic trajectories during the pandemic. State and local epidemiologists can use this tool to integrate mobility insights with other pandemic surveillance indicators to help assess the impacts of policy by geographic regions and distill these data and results into recommendations for policy makers. We have also demonstrated that the data, analyses and setting-specific information can provide epidemiologically relevant insights such as those uncovered around urban migration events. In addition, our framework could be useful beyond the current COVID-19 pandemic where understanding human mobility and behavior would help optimize interventions for natural disasters, seasonal movements or even a new pandemic. We believe the research in the Article will provide insights for epidemiologists and policy makers as they consider more modern, optimized and targeted intervention strategies in public health.

## Methods

### SafeGraph mobility data

We obtained mobility data from SafeGraph. SafeGraph aggregates mobile device Global Positioning System data from various sources and produces anonymized datasets aggregated at the CBG level. In this study, we estimate the number of people who stay at home each day by dividing the number of mobile devices that do not leave their homes by the total number of devices in each CBG (that is, completely_home_device_count divided by the device_count) (Supplementary Fig. 20)^7^. We used data covering 117 days of mobility, starting from 23 February 2020. Figure 1b illustrates this daily stay-at-home fraction for five CBGs.

SafeGraph defines a person’s ‘home’ to be the location where the mobile device is detected most at night (from 18:00 to 7:00) over a six-week period^28^. Location is defined at the Geohash-7 level (~153 m by 153 m). If a person spends enough time in a new location, that new location can become the device’s ‘home’. We use the most recently released versions of the SafeGraph social distancing data, which is version 2.0 (‘v2’) for dates before 10 May 2020 and version 2.1 (‘v2.1’) for later dates^28^. With v2.1, SafeGraph began using ‘rolling windows’ to assign the home census block group of devices instead of batch-updating only at the first of each month.

We define the daily proportion of devices seen near their homes to be the number of devices in each CBG detected in their home CBG (destination_cbg = origin_census_block_group) divided by the number of devices associated with the CBG (device_count). The proportion of devices that are only detected away from their homes each day is 1 minus this proportion; see Supplementary Fig. 18 for an example of this metric from the 2020 wildfires in Oregon.

### Census and geographic data

We obtained US population data from the 2018 American Community Survey (ACS) product of the US Census Bureau, accessed using the R package tidycensus^36^. We used table B01001 for total population size and population by age estimates by CBG, table B19013 for median household income, table B14002 for the number currently enrolled in college, table B25008 for renter- versus owner-occupied housing units and tables B07201, B07202 and B07203 for ‘geographic mobility’ (living in the same house as last year). We computed a CBG’s population density by dividing the 2018 population estimate by the land area of the CBG as reported by the cartographic boundary files.

The US Census provides cartographic boundary files, which define simplified shapes of geographic entities designed for plotting. The detailed map of Seattle was generated using ESRI’s World Topographic Map^37^ obtained using R’s OpenStreetMap package^38^. To map CBGs to zip code tabulation area (ZCTA), we assigned each CBG to the ZCTA that contained the largest portion of its geographic area.

### COVID-19 test and case data

Data on COVID tests in Washington state were provided by Washington state Department of Health through the Washington Disease Reporting System (WDRS). Data were aggregated by zip code and specimen collection date. We use the WDRS test data compiled on 16 December 2020.

### Manifold learning and nonlinear dimensionality reduction

Laplacian eigenmaps are a nonlinear manifold learning method that can identify a low-dimensional embedding that optimally preserves local structure of a high-dimensional data manifold^24^. Using the SafeGraph time-series data (section ‘SafeGraph mobility data’), we construct a mobility data matrix for each state. Each state’s data matrix has 117 columns (days of mobility data), but a different number of rows depending on the number of census block groups (Supplementary Table 3). Figure 1c illustrates the aggregation of mobility time-series into a data matrix for Washington state. To construct an *m*-dimensional embedding, the method uses *m* eigenvectors of the nearest-neighbors graph Laplacian corresponding to the smallest non-zero eigenvalues. The resulting embedding is optimal in the sense that ‘close’ data points on the original manifold are represented by points that are close in the *m*-dimensional Euclidean embedding space^24^. We also investigated a wide variety of other nonlinear dimensionality reduction techniques (Supplementary Section 2). Most notably, we also investigated diffusion maps, which are a generalization of Laplacian Eigenmaps that do not assume the underlying data distribution is uniform^22^; similarly, we also explored recently developed extensions with variable-bandwidth kernels, which do not rely on the underlying manifold being compact^26,27^ (Supplementary Section 2). The Laplacian Eigenmaps algorithm was implemented using the SpectralEmbedding function from the sklearn.manifold module of the scikit-learn package^39^ in Python 3. In this work, we used 50 neighbors for the n_neighbors parameter. Varying the number of neighbors between 20 and 50 did not notably change the Laplacian Eigenmap embedding for Washington state (Supplementary Section 2).

The optimal effective dimensionality of the embedding was identified using the trustworthiness metric^40^, which captures the extent to which a dimensionality reduction technique retains the local structure of the original data manifold from the higher-dimensional space. Trustworthiness was computed as a function of the Laplacian Eigenmap embedding dimensionality; a knee-point detection algorithm was then used to identify the optimal number of dimensions. Supplementary Section 2 provides a detailed description of this analysis for each state. To implement the trustworthiness metric, we used the function trustworthiness from sklearn.manifold of the scikit-learn package^39^ in Python 3 with default parameters (five neighbors, to capture the local structure). For the knee-point detection, we used the Kneedle algorithm implemented in the kneed package^41^. The computational codes to generate all results and figures in this Article are publicly available^42^.

### GMM clustering and uncertainty quantification

To interpret the low-dimensional structure revealed by the manifold learning, we apply GMM clustering^43^. The GMM is a latent variable model that assumes that the data have subpopulations or clusters that follow Gaussian distributions with parameters governing the centroid location and covariance structure of each cluster. GMMs were implemented using the mclust^44^ package of R (v4.0^45^). We leverage the probabilistic formulation of the GMM model as a natural way to quantify uncertainty of the cluster assignment. More specifically, the Gaussian mixture model assumes that the data have *K* subpopulations that follow Gaussian distributions with parameters *μ*_*k*_ and *Σ*_*k*_, respectively and that a latent discrete variable *z*_*i*_ ∈ {1, …, *K*} controls from which subpopulation a data point *x*_*i*_ comes^43^. If *π* corresponds to the probability mass function of *z*_*i*_, then the GMM model has the form

$$P({x}_{i}| \theta )=\mathop{\sum }\limits_{k=1}^{K}{\pi }_{k}{{{\mathcal{N}}}}({x}_{i}| {\mu }_{k},{{{\Sigma }}}_{k}),$$

where *θ* stands for the set of all parameters of the model and ${{{\mathcal{N}}}}({x}_{i}| {\mu }_{k},{{{\varSigma }}}_{k})$ is the probability density function of a normal distribution. We note that GMM could be seen as a generalization of the famous *K*-means clustering algorithm^46^.

The probabilistic formulation of the GMM model provides a natural way to quantify uncertainty of the cluster assignment. Using Bayes’ theorem, the posterior probability *P*(*z*_*i*_ = *k*∣*x*_*i*_, *θ*) that point *x*_*i*_ belongs to cluster *k* can be computed as follows:

$$P({z}_{i}=k| {x}_{i},\theta )=\frac{P({z}_{i}=k| \theta )P({x}_{i}| {z}_{i}=k,\theta )}{\mathop{\sum }\nolimits_{k^{\prime} = 1}^{K}P({z}_{i}=k^{\prime} | \theta )P({x}_{i}| {z}_{i}=k^{\prime} ,\theta )}=\frac{{\pi }_{k}{{{\mathcal{N}}}}({x}_{i}| {\mu }_{k},{{{\Sigma }}}_{k})}{\mathop{\sum }\nolimits_{k^{\prime} = 1}^{K}{\pi }_{k^{\prime} }{{{\mathcal{N}}}}({x}_{i}| \mu ^{\prime} ,{{\Sigma }}^{\prime} )}.$$

Then, the amount of uncertainty *ϵ*_*i*_ in the cluster assignment of point *x*_*i*_ could be computed as

$${\epsilon }_{i}=1-\mathop{\max }\limits_{k}P({z}_{i}=k| {x}_{i},\theta ).$$

We note that the above formula assumes that the cluster assignment is computed as

$${z}_{i}^{* }=\arg \mathop{\max }\limits_{k}P({z}_{i}=k| {x}_{i},\theta ).$$

We used BIC to identify the optimal number of GMM components^44,47,48^. BIC is based on a penalized form of the log-likelihood. As the likelihood increases with the addition of more components, a penalty term for the number of estimated parameters is subtracted from the log-likelihood^44^. To find the optimal number of GMM components, we applied knee-point detection to the BIC curve for each state (Supplementary Fig. 9). Note that in the mclust implementation, higher BIC values correspond to better models. The optimal number of clusters turned out to be four for Washington, Texas and California and five for Georgia. We used five clusters for every state in the main text for consistency across the states and to leverage optimal results for Georgia.

### Statistical testing

To test the difference between clusters in the speed at which CBGs increased their stay-at-home behavior, we used the Kolmogorov–Smirnov^49,50^ test as implemented in the kstest function of the scipy.stats package in Python 3; here, we assume these speeds are drawn from a normal distribution. To determine the significance of trends of covariates associated with CBGs in clusters identified by the GMM, we used jonckheere.test from the clinfun package^51^ in R using 1,000 permutations and assuming decreasing trends from cluster A to cluster D (Extended Data Fig. 1). The Jonckheere–Terpstra test’s null hypothesis is that covariate values are from the same distribution across clusters and the alternate is that the median covariate values are in an a priori order (that is, are increasing or decreasing from cluster A to cluster D).

### Supplementary information

Supplementary Information Supplementary Information, Figs. 1–21, Discussion and Tables 1–3.

Peer Review Information

### Source data

Source Data Fig. 1 Source information and data to reproduce each panel for Fig. 1.

Source Data Fig. 2 Source information and data to reproduce each panel for Fig. 2.

Source Data Fig. 3 Source information and data to reproduce each panel for Fig. 3.

Source Data Fig. 4 Source information and data to reproduce each panel for Fig. 4.

Source Data Extended Data Fig. 1 Source information and data to reproduce each panel for Extended Data Fig. 1.

## Data Availability

The SafeGraph mobility data used in our analysis can be obtained free of charge for non-commercial use by joining their COVID-19 Data Consortium at https://www.safegraph.com/covid-19-data-consortium. US population data from the 2018 American Community Survey (ACS) product of the US Census Bureau are publicly available; we accessed the data using the R package for the 2018 data tidycensus^36^. The 2019 shapefiles for both CBGs and ZCTAs are publicly available and were downloaded from the US Census Bureau website https://www.census.gov/geographies/mapping-files/time-series/geo/cartographic-boundary.html. COVID-19 testing data were collected as part of routine public health surveillance by the Washington State Department of Health through the Washington Disease Reporting System (WDRS; contact I. Corbridge (ian.corbridge@doh.wa.gov)), for researchers who meet the criteria for access to confidential data.

## Code availability

All computer code required to generate these results is publicly available^42,52^.
