# Supplementary material for: Insights into population behavior during the COVID-19 pandemic from cell phone mobility data and manifold learning
Source: Nat Comput Sci. 2021 Sep 22;1(9):588–97. doi: 10.1038/s43588-021-00125-9 (PMC10766515; doi:10.1038/s43588-021-00125-9)
Supplement: Supplementary file 1 — Supplementary Information, Figs. 1–21, Discussion and Tables 1–3. [file 43588_2021_125_MOESM1_ESM.pdf]

nature portfolio

https://doi.org/10.1038/s43588-021-00125-9

Supplementary information

Insights into population behavior during the COVID-19 pandemic from cell phone mobility data and manifold learning

In the format provided by the authors and unedited

SUPPLEMENTARY INFORMATION: Insights into population behavior during the COVID-19 pandemic from cell phone mobility data and manifold learning

Roman Levin[1], Dennis L. Chao[2], Edward A. Wenger[2], and Joshua L. Proctor[2]
[1] Department of Applied Mathematics, University of Washington, Seattle, WA 98195, United States
[2]Institute for Disease Modeling, Bill and Melinda Gates Foundation, WA 98109, United States

1 Linear dimensionality reduction and clustering

The singular value decomposition (SVD) is a standard linear matrix factorization technique that can be used to reduce the dimensionality of a data matrix [1, 2]. Using the SafeGraph time-series data, we construct a mobility data matrix for each state. Each state's data matrix has 117 columns (days of mobility data), but a different number of rows depending on the number of census block groups. For data matrix normalization, columns are mean subtracted. We perform a standard SVD to find a reduced order set of singular vectors and values for dimensionality reduction. The computational code to generate all results and figures in this article are publicly available [3]. Supplementary Figure 1 illustrates that more than 60 singular vectors (principal components) are required to reach 90% of the explained variance. This singular value distribution suggests a marginal benefit from linear dimensionality reduction. Moreover, applying a GMM to the data in the transformed space, we obtained highly uncertain GMM cluster assignments (see Supplementary Table 1 for uncertainty quantification). Supplementary Figure 1 provides a 3D visualization of clustering in the first three singular vectors. This provides an illustration for why cluster assignment is uncertain and changes depending on the model initialization. Linear dimensionality reduction and GMM clustering is not an effective method for the mobility dataset motivating the use of manifold learning methods.

0%	25%	50%	75%	100%
3.38e-08	1.91e-01	3.53e-01	4.97e-01	7.46e-01

Supplementary Table 1: Quartiles of the cluster assignment uncertainty based on a GMM with 11 clusters and the SVD with 8 modes.

2 Nonlinear Dimensionality Reduction Methods

We investigated a variety of nonlinear dimensionality reduction methods: t-SNE [4], locally linear embedding [5], Isomap [6], Laplacian Eigenmaps [7], local Tangent Space alignment [8], multidimensional scaling [9], and Diffusion Maps with fixed and variable bandwidth kernels [10, 11, 12]. We found that Laplacian Eigenmaps, Locally Linear Embedding, Isomap, and Diffusion maps reduced the dimensionality of the data and identified a consistent tubular dense structure in the data (Supplementary Figure 2). Supplementary Figure 2 and 3 presents 3D representations of low-dimensional embeddings for each of the methods on the example of Washington State.

Method	25%	50%	75%	100%
Laplacian Eigenmaps	0	0	1.11e-15	4.99e-01
Locally Linear Embedding	1.65e-06	6.16e-04	4.30e-02	6.18e-01
Isomap	4.79e-08	5.90e-05	4.32e-03	4.97e-01

Supplementary Table 2: **Quartiles of uncertainty of the cluster assignment based on GMM with 5 clusters.**

As described in Methods of the main manuscript, we chose the optimal embedding dimensionality for Laplacian Eigenmaps based on the knee-point of the trustworthiness metric as a function of number of dimensions. All five methods qualitatively agree on the intrinsic dimensionality of the mobility data with the optimal dimensionality identified to be between 14 and 18 dimensions (see Figure 4 for the trustworthiness metric plots for Washington State). These methods capture the structure of the data well and produce cluster assignments with significantly lower associated uncertainty than linear dimensionality reduction (see Supplementary Table 2). The exception is for the variable bandwidth Diffusion Maps embedding and GMM; depending on the input parameters k for number of neighbors used and ϵ for the constant kernel bandwidth, the CBG membership in cluster E is more variable. Supplementary Figure 3 visualizes the difference for the embedding, CBG membership, and aggregate stay-at-home time series for Washington state when the number of neighbors used varies. There is a difference in membership, but at $k = 100$ the variable bandwidth kernel Diffusion Maps with GMM is similar to the other embedding methods with GMM. We used guidance from [12] and the `DiffusionMap.from_sklearn` function from `diffusion_map` module of the Python package `pydiffmap` version 0.2.0.1 to choose parameters and investigate sensitivity [13]. Following [12], for Diffusion Maps with fixed bandwidth kernel we used $k = 50$ for the number of neighbors, $\epsilon = 0.5$ for the kernel bandwidth coefficient, $\alpha = 1$ for the debiasing term, and found the optimal dimensionality to be $d = 15$. For Diffusion Maps with variable bandwidth kernel, we used $\epsilon = 2e-4$, $\alpha = 1/2 - d/4$, $\beta = -1/2$, $d = 15$, and tried $k = 100$ and $k = 250$. The quantitative similarities across methods provides confidence in the robustness of these results given the differing underlying assumptions for the data distribution. Both methods, fixed and variable bandwidth kernel Diffusion Maps, do not rely on uniform sampling of the data distribution. Also, the variable bandwidth kernel Diffusion map method does not require compactness of the manifold [11, 12]. In our numerical experiments, the GMM has difficulty identifying cluster E with the embedding appearing more robust. We see this as an important research direction to investigate in future work both for analyzing mobility time-series from smartphones and understanding how to select parameters for these nonlinear manifold learning methodologies.

We selected Laplacian Eigenmaps as the primary methodology because it produced the least uncertain GMM cluster assignment and the clustering was robust to perturbations in the method's single hyperparameter, `n_neighbors` (Supplementary Figure 5). As mentioned earlier, the other methods produce quantitative and qualitatively similar results. Trustworthiness was computed as a function of the Laplacian Eigenmap embedding dimensionality; a knee-point detection algorithm was then used to identify the optimal number of dimensions. Supplementary Figure 6 shows the optimal Laplacian eigenmaps dimensionality is 14 for every state. Note that California is different in that there are two possible knee points: one that is consistent with other states at 14 and another at 44 dimensions. We compared clustering results for these two knee points (Supplementary Figure 7) and identify that the cluster assignments are similar. Therefore, in our main analysis we used 14D Laplacian eigenmap embedding for California.

3 Robustness of GMM fitting

The underlying objective function for the standard implementation of a Gaussian mixture model is not convex. We ensured that the GMM clustering produced consistent and robust results by re-initializing the fitting algorithm many times. As an example, Supplementary Figure 8 illustrates the GMM fit for six different initializations. We observe that the results are qualitatively similar with the main difference that the sparse region sometimes is identified as a separate cluster or divided into two clusters.

4 Altering the Number of Clusters and Continuous Colormap

While the optimal number of clusters for Washington, Texas, and California was 4 (based on knee-point detection in BIC, we chose 5 as the number of clusters for every state in our main analysis. Allowing for more clusters provides more granular information within urban areas while maintaining consistency with the 4 cluster model. Supplementary Figure 10 presents the clustering results with the optimal number of clusters for every state. Note that Figure 2 in the main manuscript provides more granular information for Washington, Texas, and California. Increasing the number of clusters beyond the optimal results in a finer partitioning of the embedding (Supplementary Figure 11).

We also demonstrate that by modeling the data with a single dimensional parameter in the nonlinear embedding along the dense tubular manifold matches the intuition provided by increasing the number of clusters for the GMM. For example, we constructed a single dimensional phase variable along the manifold based on the cosine similarity of the data points in the 2D nonlinear embedding space. The result is an even smoother transition across urban, periurban, suburban, and rural areas consistent across all four states, see Supplementary Figure 12.

5 Clustering in metropolitan areas: Georgia and California

Supplementary Figures 13 and 14 present the clustering for metropolitan areas in Georgia and California, respectively.

6 Response Speed Distributions

The CBG clustering and average time-series by cluster also indicate that the change of behavior over time is different across clusters before April. The speed at which CBGs increased their stay-at-home behavior during a transition period between March and April (quantified by the slope of a linear fit of the CBG mobility time series during the transition period) is directly correlated with CBG cluster assignment. Moreover, the distributions of that speed are statistically significantly different: for every pair of clusters, we were able to reject the null hypothesis that the speed distributions were the same at the significance level ($p < 0.01$) using Kolmogorov-Smirnov test. This is also directly evident by looking at this time period and the average stay-at-home trends by cluster (main article Figure 2). For example, the CBGs from the least mobile cluster A also increased their staying-at-home level the fastest.

We quantified the speed at which CBGs increased their stay-at-home behavior in response to the pandemic during a transition period between March and April (more specifically, March 10 – March 31) by the slope of a linear fit of the CBG mobility time series during the transition period

(Supplementary Figure 15). Supplementary Figure 15 shows that the response speed distributions are directly correlated with CBG cluster assignment.

7 Comparison of SafeGraph to Google and Facebook's data

We compared the SafeGraph stay-at-home metric to publicly accessible data from Google and Facebook. Google offers aggregated and annonymized mobility data, similar to that used in Google maps, which includes a measure of the relative change in duration of residential stays [14]. Similarly, Facebook provides movement range maps that includes a measure of the positive proportion of users staying put within a single location [15]. Both Facebook and Google data is only available at the county level. We aggregated the Safegraph data to the county level to compare across data providers. Supplementary Figure 21 shows how the three data sources compare for two counties in Washington state. For both counties, the qualitative levels and trends are similar across the three data providers at the county level; this matches observations by Weill et al. [16]. For the analyses and modeling in this article, we leverage the SafeGraph data at the census block group level.

8 Additional Discussion on links to COVID Epidemiology

The data-driven approach detected due the sharp changes in the time-series – SafeGraph had been updating the "home" locations of mobile devices at the beginning of each month before May 2020, introducing monthly discontinuities in the stay-at-home time series in certain CBGs. This was an unexpected finding that we would have missed if not for our data-driven approach. Notably, we have identified features in the SafeGraph stay-at-home data that strongly suggests a mass migration out of several major metropolitan areas, especially in CBGs that have high proportions of young adults, renters, or students. The closure of college of campuses and widespread job losses in March and April led many, especially young adults, to move [17, 18], and there was widespread urban to rural migration in anticipation of lockdowns [19]. Moreover, the map presented in Extended Data Figure 2 also matches our own intuition of where students of the University of Washington live, both adjacent to the university as well as more distant rental housing along bike and metro commuting lines (all authors of this article live in the greater Seattle area). Similarly, the high-migration census block groups identified near South Lake Union tends toward a younger, professional population working at technology companies such as Amazon, and CBGs on the waterfront with high income populations in nearby cities such as Bellevue and Kirkland have a similar outward migration trend.

Identifying the population that moved early in the pandemic is a direct consequence of using a data-driven, equation-free approach. The approach has been integral to revealing the heterogeneity, but also the consistency, of mobility patterns across California, Georgia, Texas, and California; it has enabled a multi-scale geographic perspective on behavior allowing insights at the state, urban-rural, peri-urban, and suburban scale. Recent efforts have also utilized clustering of mobility time-series data specifically for analyzing SafeGraph stay-at-home data in Atlanta [20]. Our approach, though, is substantially broader in scope; identifying the low-dimensional embedding of the data enables a characterization of the geometric structure and the relatedness of each CBG mobility behavior. Moreover, we found utilizing nonlinear dimensionality reduction techniques such as Diffusion Maps for analyzing mobility time-series data is essential (Supplementary Section refss:svd) mirroring recent developments from dynamical systems focused on the development of equation-free methods for analyzing measurement data collected from com-

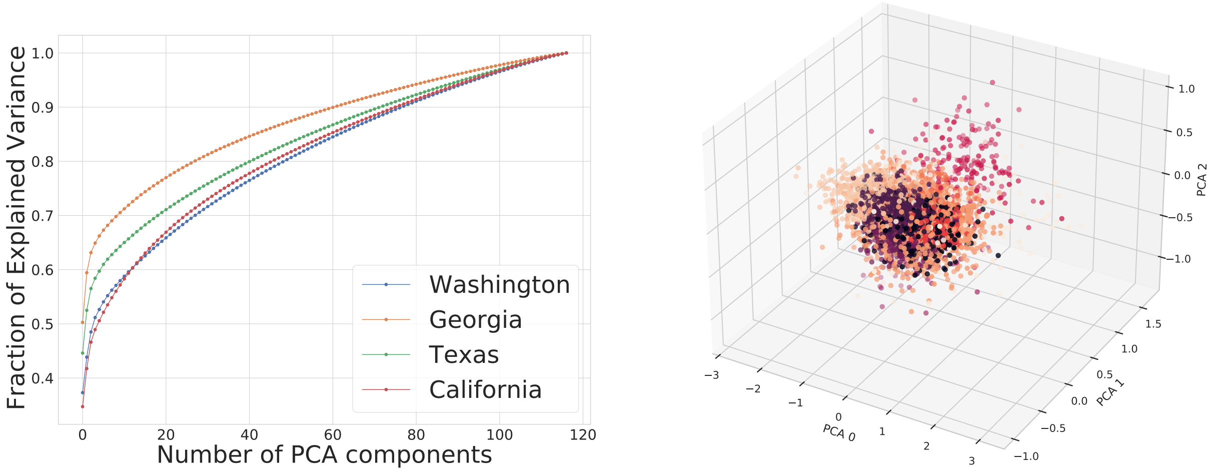

Supplementary Figure 1: **Linear Dimensionality Reduction Performance.** Left: fraction of explained variance vs. number of PCA components for every state. Right: 3D visualization of PCA projection of the mobility time series data for Washington State with 11 clusters highlighted in color. Clustering was done in 8D PCA space.

plex systems [10, 11, 12]. We have also leveraged clustering as a tool to interpret the similarity of mobility behavior between CBGs even in the reduced nonlinear embedding; we found that clusters allowed for comparisons of mobility characteristics, generalization across four states, and also correlation with socioeconomic factors. The nonlinear embedding, however, offers a more nuanced perspective about the similarity of mobility behavior between CBGs. For example, the visualization in three dimensions and the clustering results suggests a much smoother and continuous geometric structure of relatedness for CBGs assigned to clusters A, B, C, and D. This helps frame the clustering results and socioeconomic factor correlation analysis. Further, the embedding provides a richer characterization of the underlying complexity in mobility behavior.

We find that higher-risk populations can be identified early in the pandemic using mobility data both in terms of aggregate stay-at-home behavior and urban migration events. For example, cluster E, a young and less settled population, had distinct outbreaks in late June and late September, corresponding to reported outbreaks in the University of Washington's Greek community [21, 22]. College campuses, with their young populations living in shared housing, could have epidemic dynamics different from their surrounding communities. Many colleges and universities closed their campuses in March in response to COVID-19 and thousands of young adults moved back to campuses when they re-opened in the fall, which could have fueled surges in SARS-CoV-2 transmission [23]. In addition, college athletic programs and the Greek system bring large groups of people together. Both specific epidemiological examples like this and the intuitive aggregate results linking population mobility to COVID-19 risk demonstrates the value of this mathematical approach and cell-phone mobility data.

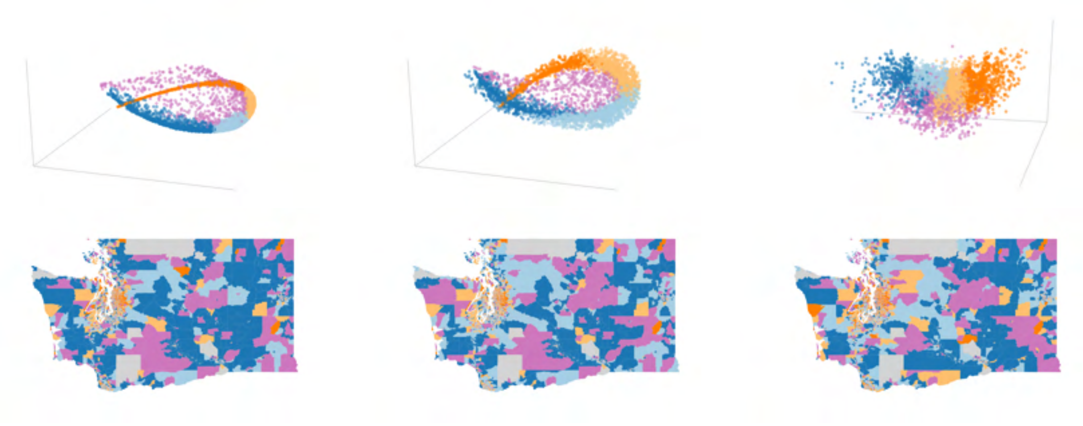

Supplementary Figure 2: **Nonlinear Dimensionality Reduction Embeddings and Corresponding Maps for Washington State.** Top Left: Laplacian Eigenmaps, Top Middle: Locally Linear Embedding, Top Right: Isomap. Bottom Left: Laplacian Eigenmaps clustering map, Bottom Middle: Locally Linear Embedding clustering map, Bottom Right: Isomap clustering map.

Supplementary Table 3: Number of census block groups (CBGs) in each cluster.

state	A	B	C	D	E	Total
California	2672	5457	7053	5319	2470	22971
Georgia	1577	1149	1177	960	647	5510
Texas	3154	3960	3687	3283	1635	15719
Washington	1120	1284	1032	674	642	4752

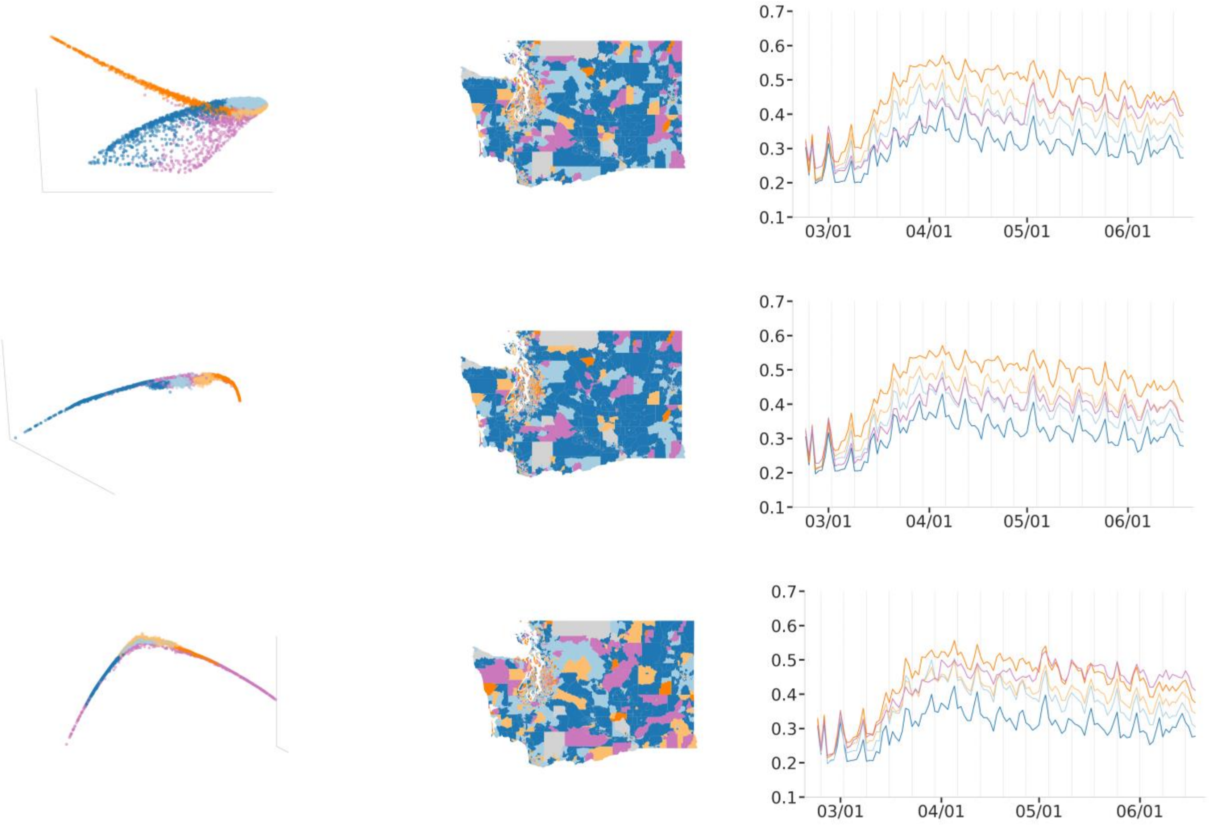

Supplementary Figure 3: **Diffusion Maps for Nonlinear Dimensionality Reduction Embeddings and Corresponding Maps for Washington State.** Top Row: Diffusion Maps with fixed bandwidth kernel and $k = 50$ number of neighbors use, $\epsilon = 0.5$ the kernel bandwidth coefficient, $\alpha = 1$ for the debiasing term, and optimal dimensionality of $d = 15$. Middle Row: Diffusion Maps with variable bandwidth kernel and $k = 100$, $\epsilon = 2e-4$, $\alpha = 1/2 - d/4$, $\beta = -1/2$, and $d = 15$. Bottom Row: Diffusion Maps with variable bandwidth kernel and $k = 250$, $\epsilon = 2e-4$, $\alpha = 1/2 - d/4$, $\beta = -1/2$, and $d = 15$.

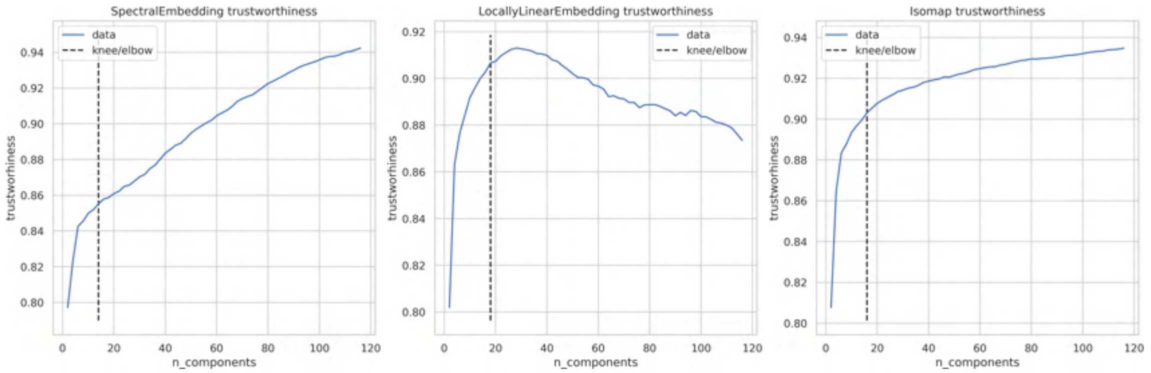

Supplementary Figure 4: **Optimal Embedding Dimensionality for Washington State.** Left: Laplacian Eigenmaps (optimal dimensionality 14), Middle: Locally Linear Embedding (optimal dimensionality 12), Right: Isomap (optimal dimensionality 16).

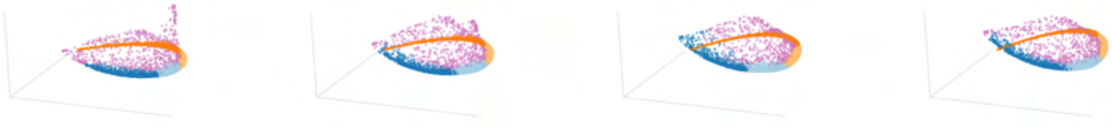

Supplementary Figure 5: **Laplacian Eigenmaps Hyperparameter Robustness.** Each panel presents a 3D Laplacian Eigenmaps embedding of Washington state mobility data for `n_neighbors` in $\{20, 30, 40, 50\}$ respectively.

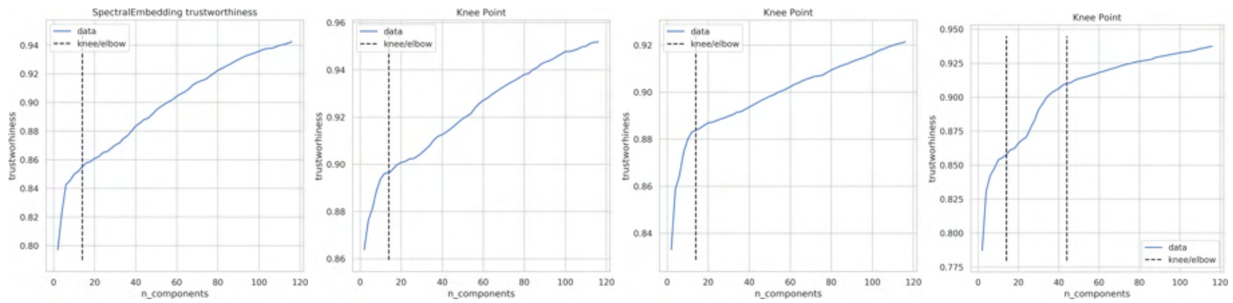

Supplementary Figure 6: **Optimal Laplacian Eigenmap Embedding Dimensionality for Every State.** Each panel presents trustworthiness vs number of Laplacian Eigenmap components for Washington (optimal dimensionality 14), Georgia (optimal dimensionality 14), Texas (optimal dimensionality 14), and California (optimal dimensionality 14 or 44) respectively.

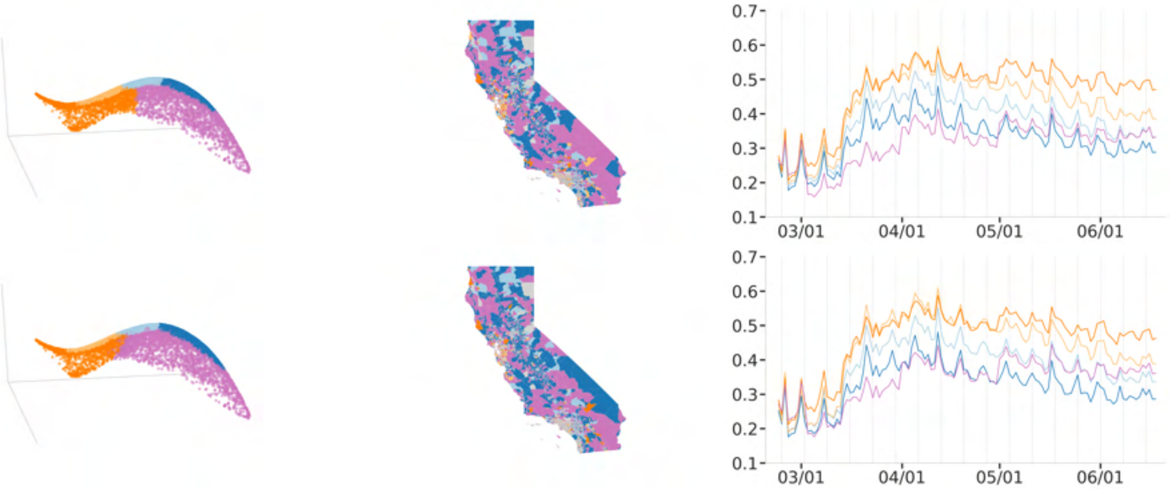

Supplementary Figure 7: **Comparison of two trustworthiness knee points for California.** Top: Clustering results using 14D Laplacian Eigenmap embedding (3D illustration of the embedding, geographic map and average mobility time series per cluster with clusters highlighted in color). Bottom: Clustering results using 44D Laplacian Eigenmap embedding (3D illustration of the embedding, geographic map and average mobility time series per cluster with clusters highlighted in color).

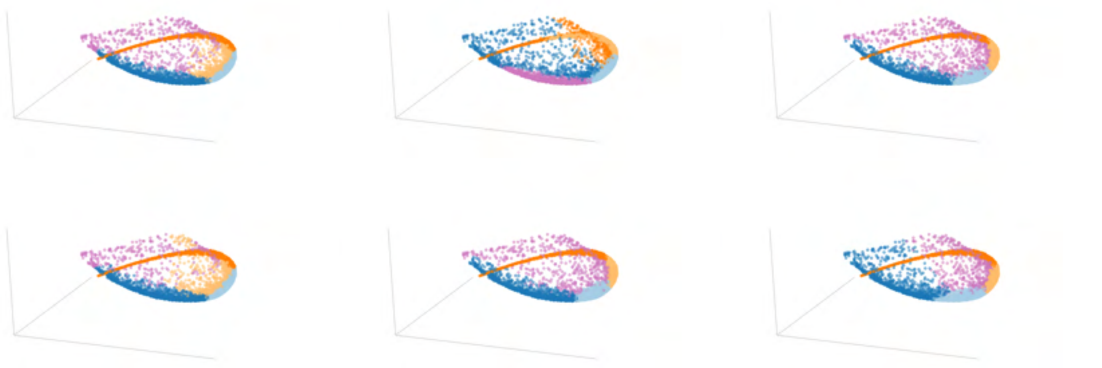

Supplementary Figure 8: **Nonconvexity robustness.** Different types of GMM clustering results obtained by refitting GMM several times.

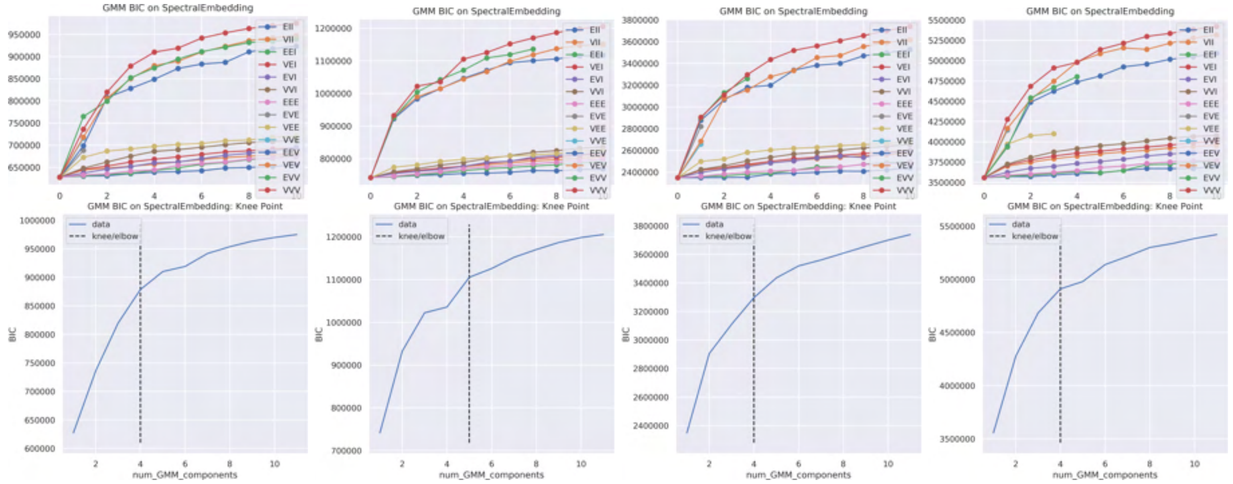

Supplementary Figure 9: **GMM model selection for every state based on BIC.** Top: BIC curves for different parametrizations of the GMM model as described in [24]. Bottom: Optimal number of GMM components identified using knee-point detection on the best BIC curve for Washington (optimal number of clusters 4), Georgia (optimal number of clusters 5), Texas (optimal number of clusters 4), and California (optimal number of clusters 4) respectively.

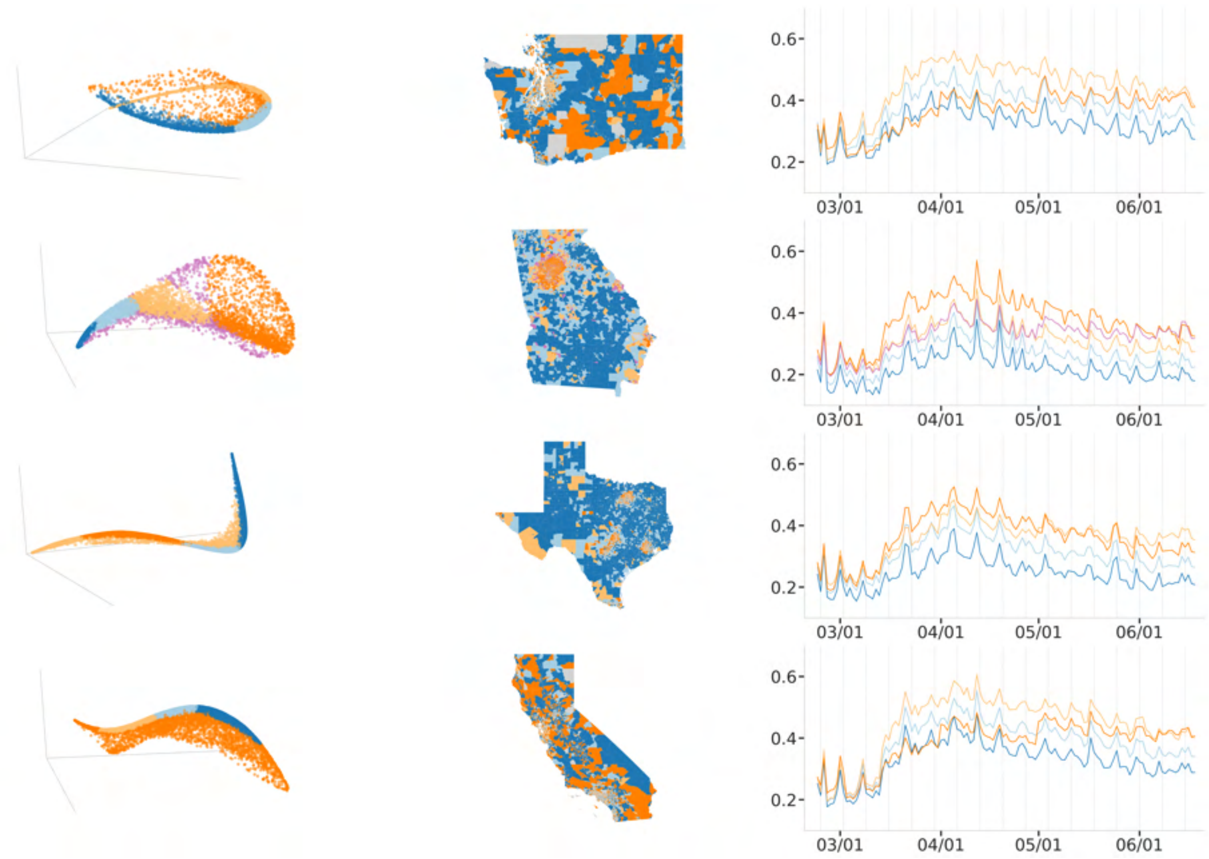

Supplementary Figure 10: **Clustering with the optimal number of clusters for every state**

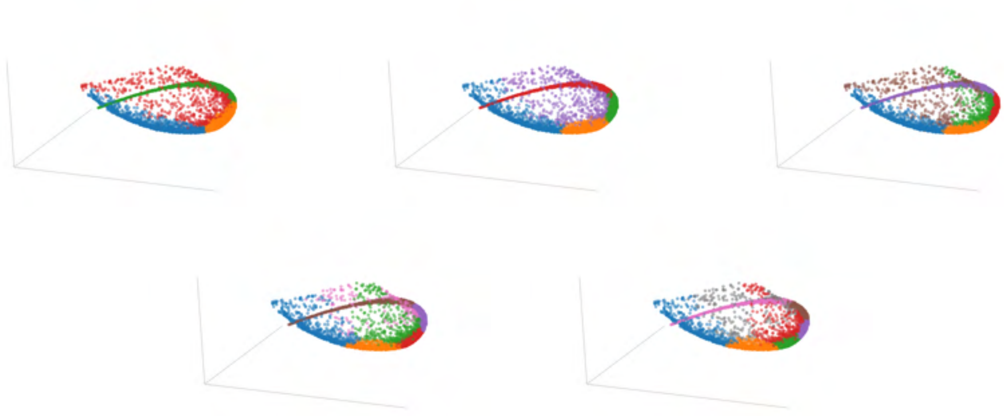

Supplementary Figure 11: **Bigger number of clusters results in finer partitioning of the embedding.** Panels present clustering for the number of clusters in $\{4, 5, 6, 7, 8\}$ respectively.

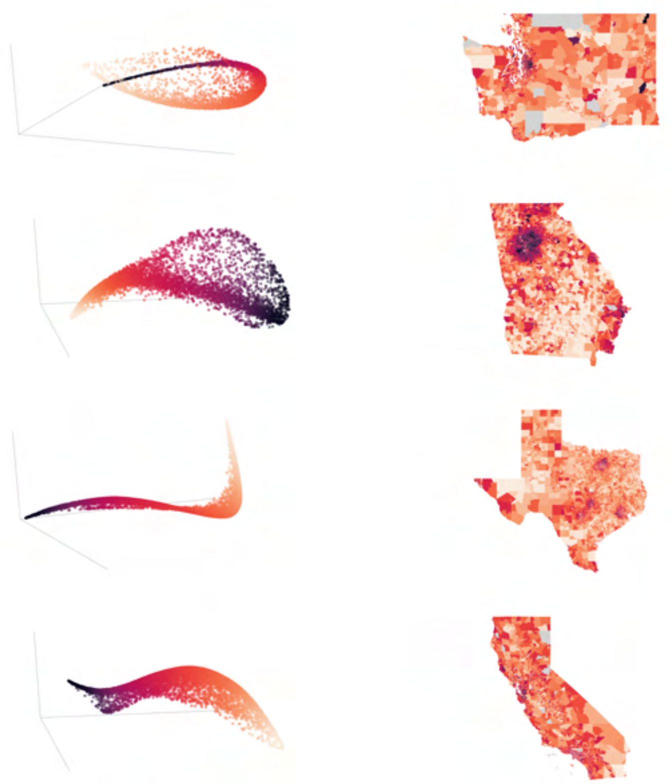

Supplementary Figure 12: **Continuous Colormap.** Smooth transition across urban, periurban, suburban, and rural areas in Washington, Georgia, Texas, and California.

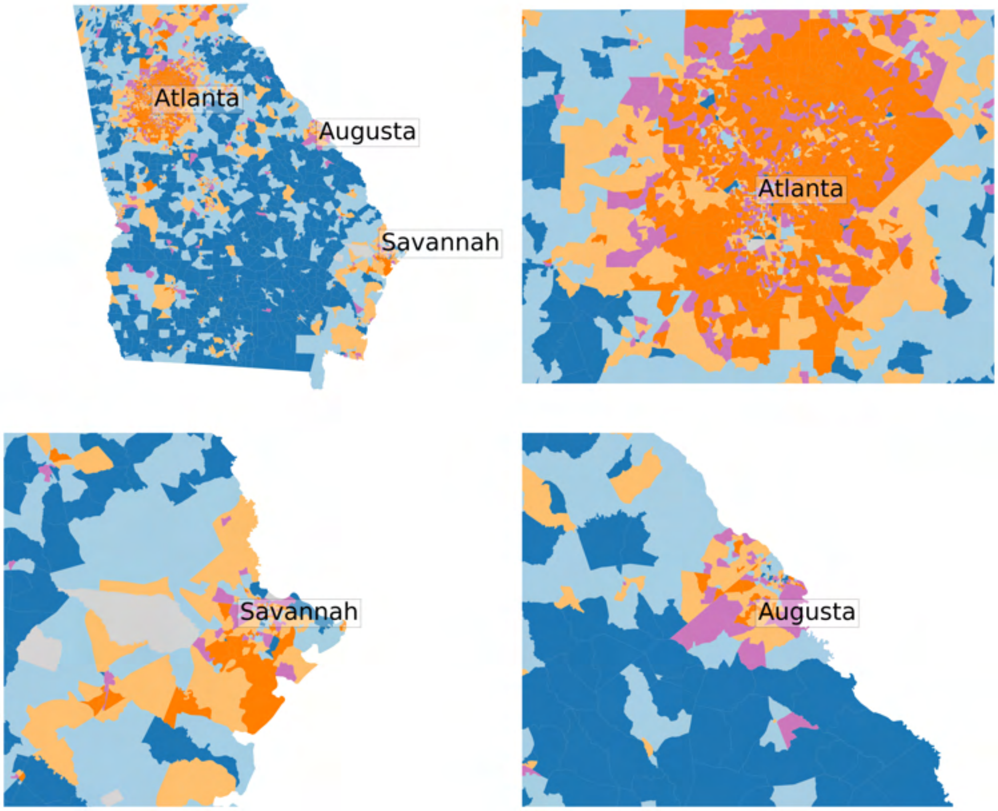

Supplementary Figure 13: **Clustering in metropolitan areas in Georgia**

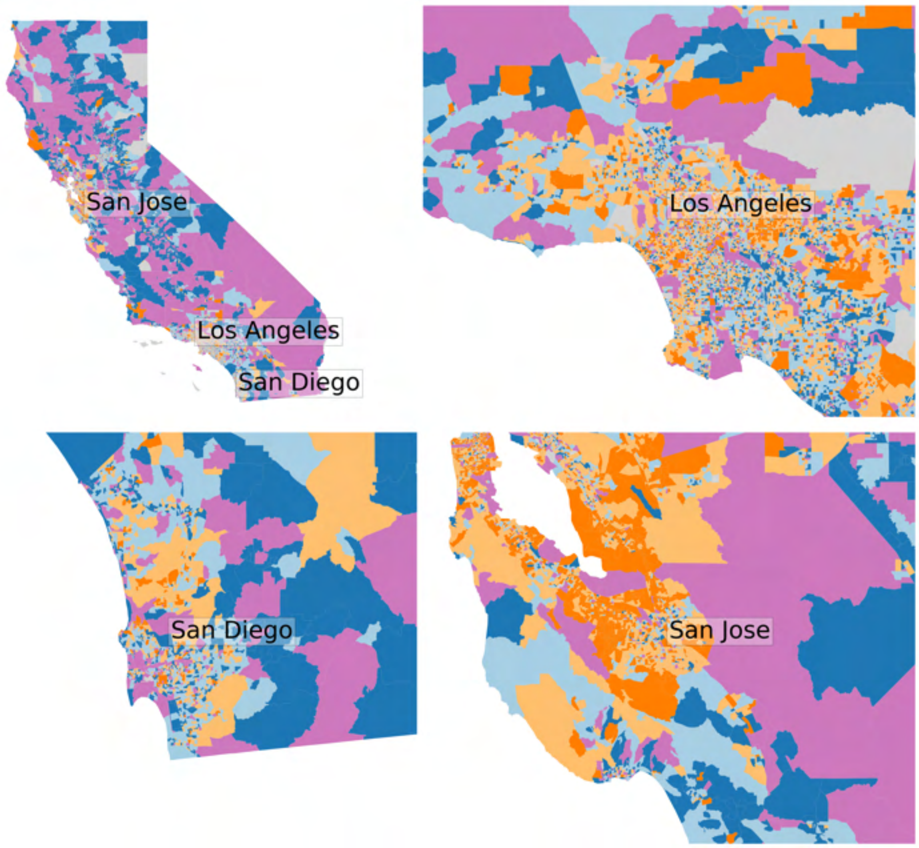

Supplementary Figure 14: **Clustering in metropolitan areas in California**

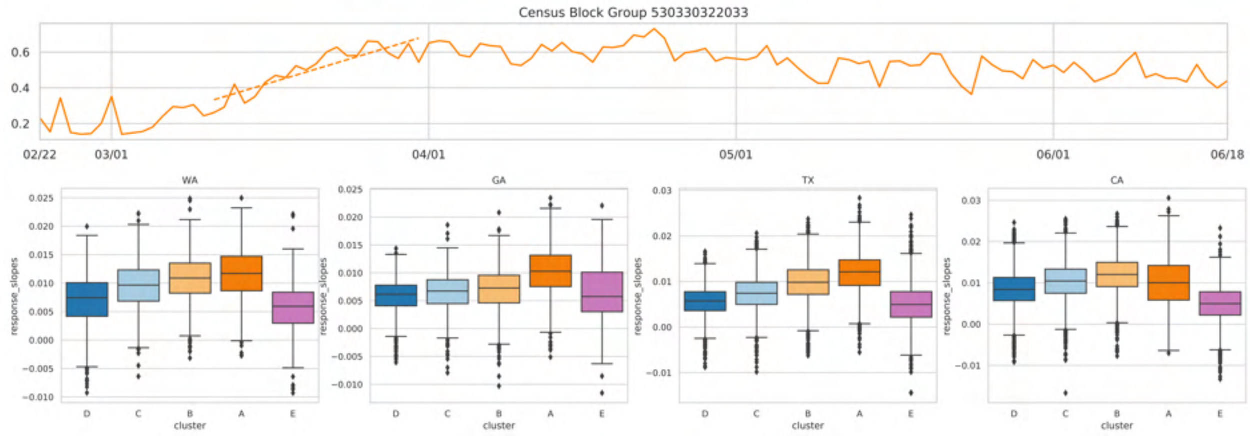

Supplementary Figure 15: **Response Speed Distributions.** Top: The response speed is quantified by the slope of a linear fit of the CBG mobility time series during the transition period of March 10 – March 31, dashed line represents that linear fit for an example CBG. Bottom: Response speed distributions for every state.

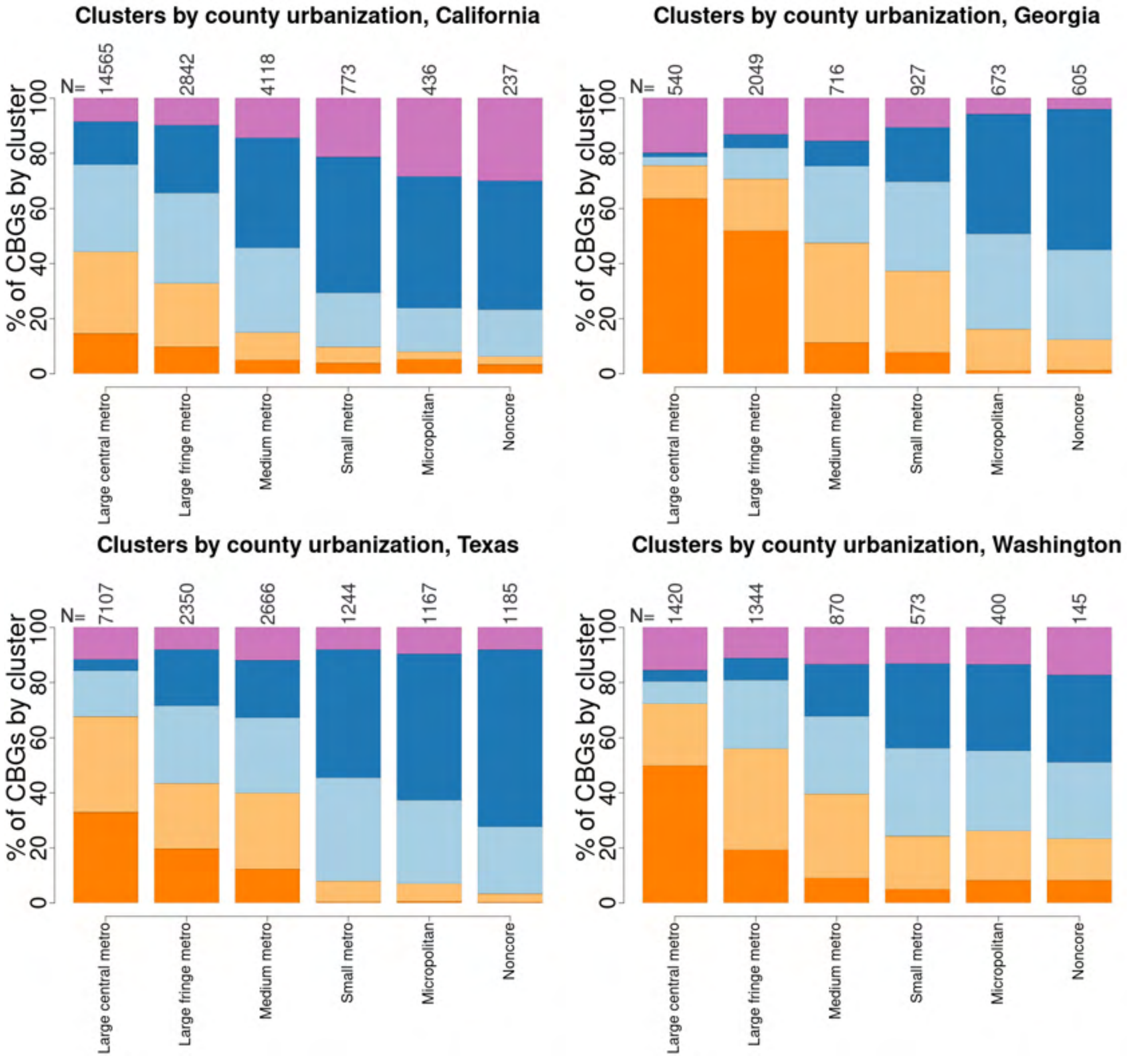

Supplementary Figure 16: **The proportion of CBGs in each cluster by urbanization.** The 2013 Urban–Rural Classification Scheme for Counties was used to categorize CBGs as "Large central metro", "Large fringe metro", "Medium metro", "Small metro", "Micropolitan", and "Noncore" (https://www.cdc.gov/nchs/data_access/urban_rural.htm). The first four categories can be considered "urban" and the last two "rural". Since classification was at the county level, all CBGs within a single county receive the same classification. The numbers of CBGs in each category are printed along the top of each panel. The proportion of CBGs in each cluster is plotted as vertically stacked bars for each bin (with cluster A in dark orange on the bottom through cluster E in purple on top).

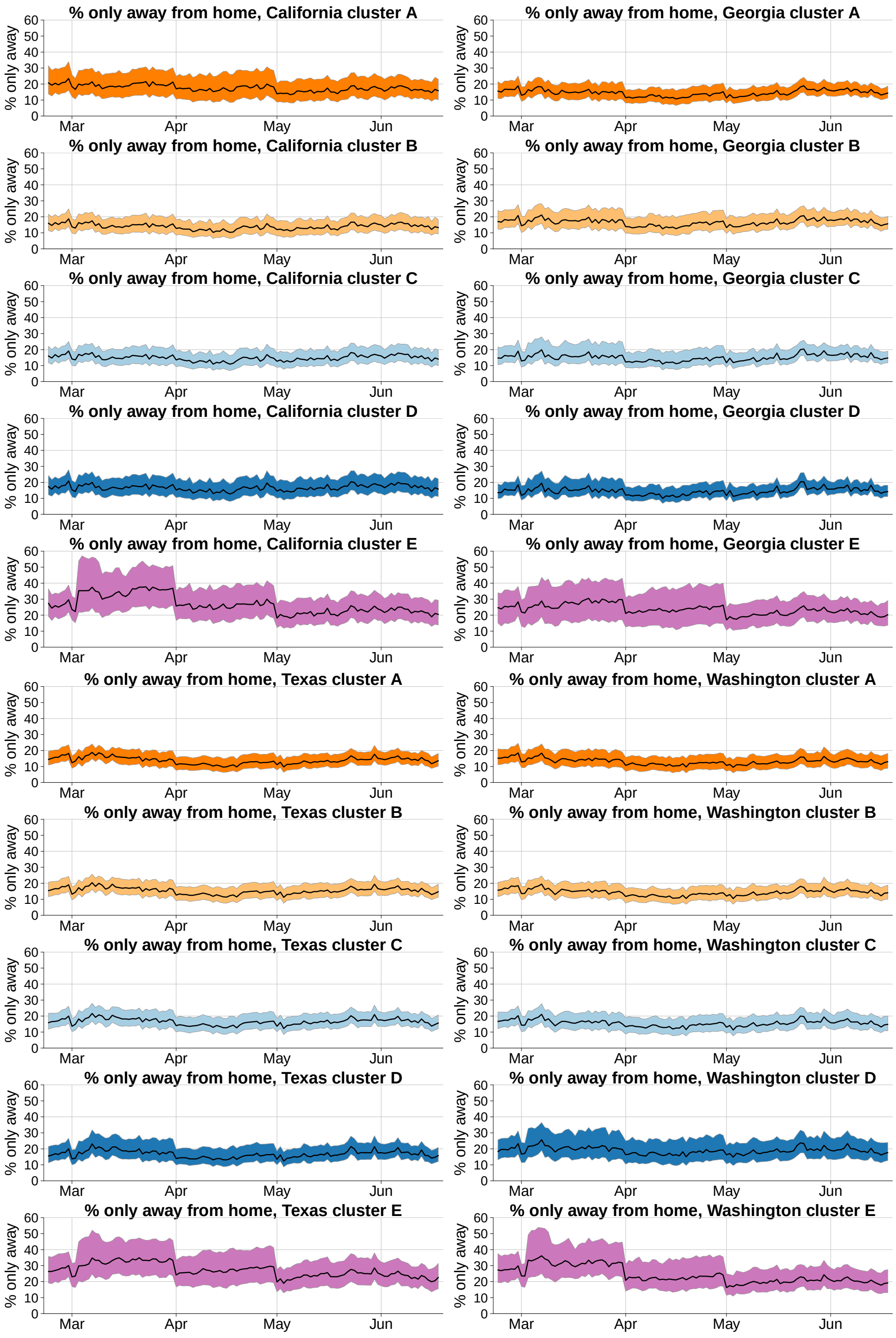

Supplementary Figure 17: **The fraction of devices that are only away from their homes each day.** The medians and inter-quartile range are shown for each cluster.

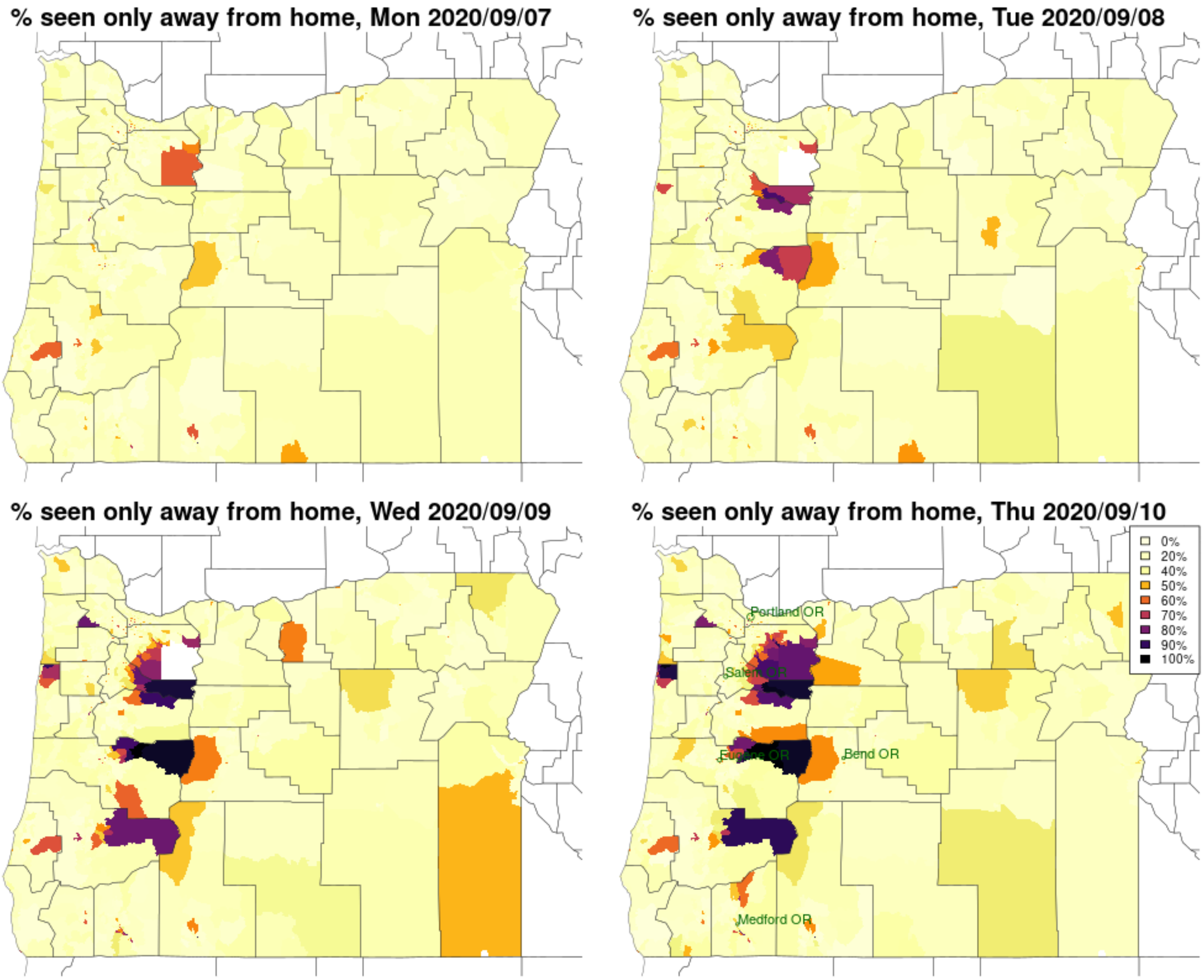

Supplementary Figure 18: **Mass evacuations can be detected as high proportions of mobile devices that are "only away from home".** Maps depict the fraction of devices that are detected only away from their "homes" each day in Oregon. A mobile device's "home" is defined as the location where it has been detected at night over the previous 6 weeks [25]. CBGs with a large proportion of mobile devices only away home are in darker colors, as indicated in the legend. The timing and location of regions where most people are away is consistent with evacuations due to wildfires that started on September 7, 2020 (https://storymaps.arcgis.com/stories/6e1e42989d1b4beb809223d5430a3750).

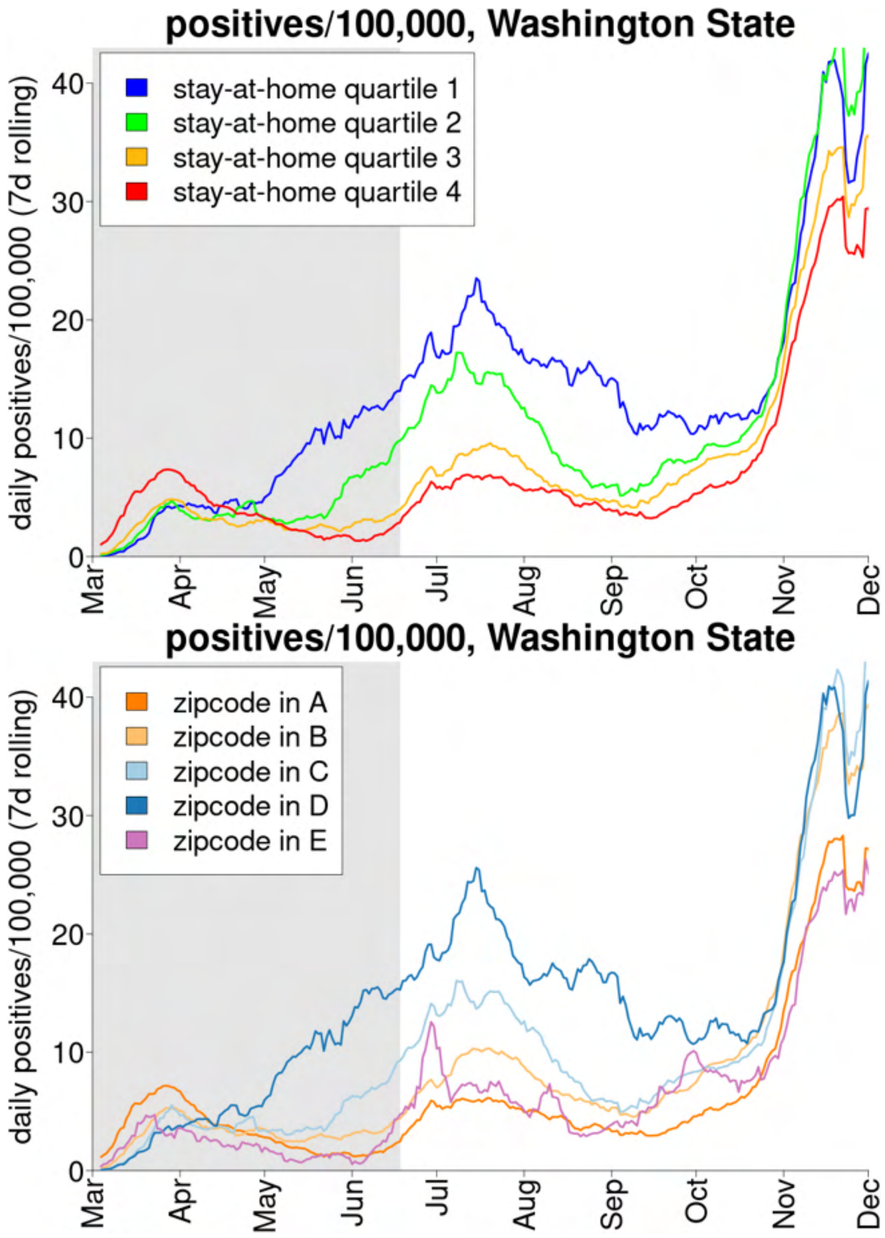

Supplementary Figure 19: **Cases in Washington State by the average fraction of devices that "stay at home."** The top panel is the number of cases per capita, stratified by the average amount people in each zip code stayed home from late February through mid-June 2020 (shaded region). The fraction of the population who stayed at home each day was computed for each CBG and averaged over time window in gray, and the zipcode-level metric was the population-weighted average of the CBGs that comprised each zipcode, as described in Methods. Quartile 1 (in blue) includes the zipcodes where people stayed home the least. The bottom panel is a copy of Figure 8B in the main text, which shows the number of cases per capita by cluster assignment. The stay-at-home quartiles in the top panel behave similarly to clusters A–D in the bottom panel, but cluster E could not be detected when behavior is averaged over time.

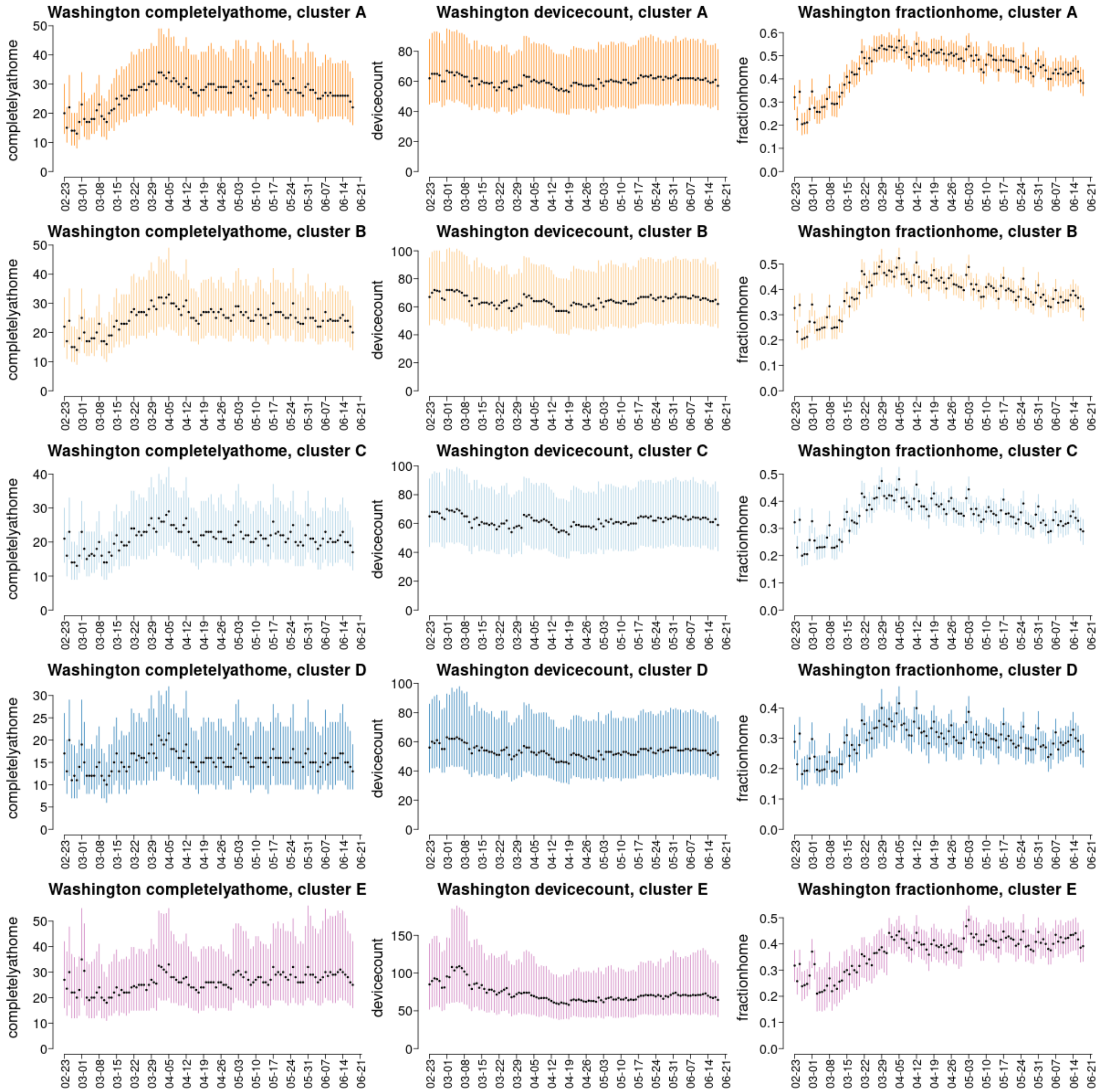

Supplementary Figure 20: **Number of mobile devices in Washington State by cluster.** The daily number of devices that were only detected at home (left column), total devices detected (middle column), and the ratio of these two values (right column) are plotted. Each row is a different cluster, from cluster A in the top row to cluster E in the bottom. The black dots are median values and shaded areas are inter-quartile ranges (the 25th to the 75th percentile of values).

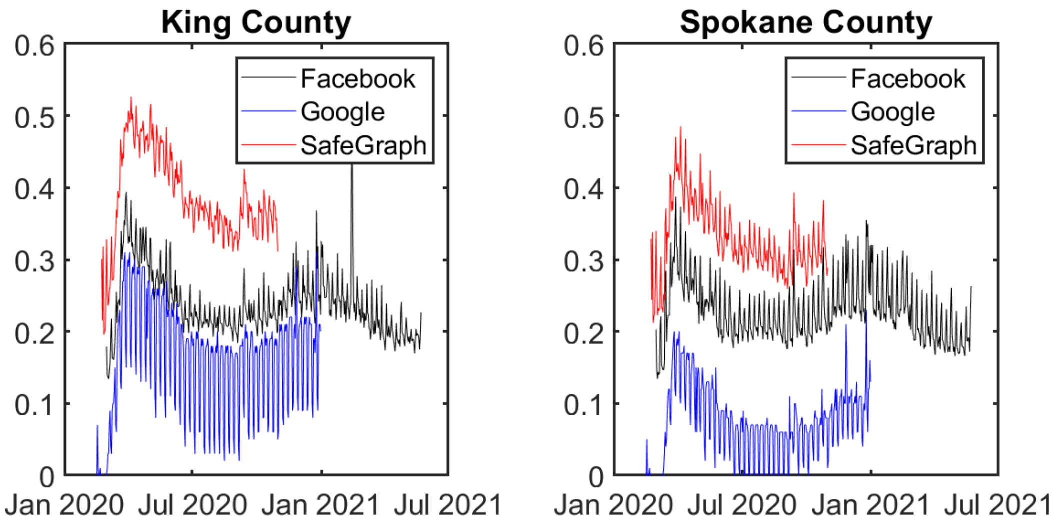

Supplementary Figure 21: Mobility data from SafeGraph, Google, and Facebook are plotted for two counties in Washington state including King and Spokane county. Each data provider offers a different metric that roughly indicates how much populations are staying near their homes at the county level.

References

[1] Pearson, K. LIII. On lines and planes of closest fit to systems of points in space. The London, Edinburgh, and Dublin Philosophical Magazine and Journal of Science **2**, 559–572 (1901).

[2] Eckart, C. & Young, G. The approximation of one matrix by another of lower rank. Psychometrika **1**, 211–218 (1936).

[3] Levin, R. Covid mobility and behavior. `https://github.com/InstituteforDiseaseModeling/covid-mobility-and-behavior` (2020).

[4] Van der Maaten, L. & Hinton, G. Visualizing data using t-sne. Journal of machine learning research **9** (2008).

[5] Roweis, S. T. & Saul, L. K. Nonlinear dimensionality reduction by locally linear embedding. science **290**, 2323–2326 (2000).

[6] Tenenbaum, J. B., De Silva, V. & Langford, J. C. A global geometric framework for nonlinear dimensionality reduction. Science **290**, 2319–2323 (2000).

[7] Belkin, M. & Niyogi, P. Laplacian eigenmaps for dimensionality reduction and data representation. Neural Computation **15**, 1373–1396 (2003).

[8] Zhang, Z. & Zha, H. Nonlinear dimension reduction via local tangent space alignment. In International Conference on Intelligent Data Engineering and Automated Learning, 477–481 (Springer, 2003).

[9] Borg, I. & Groenen, P. J. Modern multidimensional scaling: Theory and applications (Springer Science & Business Media, 2005).

[10] Coifman, R. R., Kevrekidis, I. G., Lafon, S., Maggioni, M. & Nadler, B. Diffusion maps, reduction coordinates, and low dimensional representation of stochastic systems. Multiscale Modeling & Simulation **7**, 842–864 (2008).

[11] Berry, T. & Sauer, T. Local kernels and the geometric structure of data. Applied and Computational Harmonic Analysis **40**, 439–469 (2016).

[12] Berry, T. & Harlim, J. Variable bandwidth diffusion kernels. Applied and Computational Harmonic Analysis **40**, 68–96 (2016).

[13] Banisch, R., Thiede, E. H. & Trstanova, Z. pyDiffMap (2020). URL `https://github.com/DiffusionMapsAcademics/pyDiffMap`. Python library version 0.2.0.1.

[14] Google. Covid-19 community mobility reports (2021). URL `https://www.google.com/covid19/mobility/`. Accessed on May 27, 2021.

[15] Facebook. Facebook movement range maps (2021). URL `https://dataforgood.fb.com/tools/movement-range-maps/`. Accessed on May 27, 2021.

[16] Weill, J. A., Stigler, M., Deschenes, O. & Springborn, M. R. Social distancing responses to COVID-19 emergency declarations strongly differentiated by income. Proc Natl Acad Sci U S A **117**, 19658–19660 (2020).

[17] Cohn, D. About a fifth of U.S. adults moved due to COVID-19 or know someone who did (2020). https://www.pewresearch.org/fact-tank/2020/07/06/about-a-fifth-of-u-s-adults-moved-due-to-covid-19-or-know-someone-who-did/.

[18] Fry, R., Passel, J. S. & Cohn, D. A majority of young adults in the U.S. live with their parents for the first time since the Great Depression (2020). https://www.pewresearch.org/fact-tank/2020/09/04/a-majority-of-young-adults-in-the-u-s-live-with-their-parents-for-the-first-ti

[19] Kishore, N. et al. Lockdowns result in changes in human mobility which may impact the epidemiologic dynamics of SARS-CoV-2. Sci Rep **11**, 6995 (2021).

[20] Huang, X. et al. Time-series clustering for home dwell time during COVID-19: what can we learn from it? International Journal of Geo-Information **9**, 675 (2020).

[21] UW News Staff. UW, Public Health – Seattle & King County responding to coronavirus cases in Greek system. UW News (2020). https://www.washington.edu/news/2020/06/30/uw-public-health-seattle-king-county-responding-to-coronavirus-cases-in-greek-system/.

[22] Balta, V. UW COVID-19 testing reveals few positives on campus; new outbreak in Greek community. UW News (2020). https://www.washington.edu/news/2020/10/01/uw-covid-19-testing-reveals-few-positives-on-campus-new-outbreak-in-greek-community/.

[23] The New York Times. Tracking the coronavirus at U.S. colleges and universities. The New York Times (2020). https://www.nytimes.com/interactive/2020/us/covid-college-cases-tracker.html.

[24] Scrucca, L., Fop, M., Murphy, T. B. & Raftery, A. E. mclust 5: clustering, classification and density estimation using Gaussian finite mixture models. The R Journal **8**, 289–317 (2016). URL https://doi.org/10.32614/RJ-2016-021.

[25] SafeGraph. Safegraph common nighttime location algorithm (2020). URL https://docs.safegraph.com/docs/places-manual\#section-safe-graph-common-nighttime-location-algorithm. Accessed on October 1, 2020.
