# Supplementary material for: Insights into population behavior during the COVID-19 pandemic from cell phone mobility data and manifold learning
Source: Nat Comput Sci. 2021 Sep 22;1(9):588–97. doi: 10.1038/s43588-021-00125-9 (PMC10766515; doi:10.1038/s43588-021-00125-9)
Supplement: Supplementary file 2 — Peer Review Information [file 43588_2021_125_MOESM2_ESM.pdf]

natureresearch

Peer Review Information

Journal: Nature Computational Science
Manuscript Title: Insights into population behavior during the COVID-19 pandemic from cell phone mobility data and manifold learning
Corresponding author name(s): Joshua Proctor

Editorial Notes:

**Redactions – published data**	Parts of this Peer Review File have been redacted as indicated to remove third-party material.

Reviewer Comments & Decisions:

Decision Letter, initial version:

Dear Dr Proctor,

Your manuscript "Cell phone mobility data and manifold learning: Insights into population behavior during the COVID-19 pandemic" has now been seen by 4 referees, whose comments are appended below. You will see that while they find your work of interest, they have raised points that need to be addressed before we can make a decision on publication.

The referees' reports seem to be quite clear. Naturally, we will need you to address all of the points raised.
While we ask you to address all of the points raised, the following points need to be substantially worked on:

- Please provide more details on the data generation process, as this is key to better understand the results obtained in the paper. Please also discuss the ethical implications that arise when collecting this type of data, since this can be useful to our readership.
- To better validate the robustness of the proposed methodology, and the obtained trends and results, it would be ideal to also experiment with other (similar) data, as suggested by Reviewer #1.

- Please improve the link of the obtained results to epidemiological characteristics and their biological importance.
- Comparison of the proposed method against a traditional or naive method is needed.
- Some reviewers argue that the framework, while interesting, might not be ready yet to inform policy decisions. Please better discuss this point in the paper, or change the story of the paper to better reflect what could be accomplished with the framework.
- The assumptions underlying the linear and nonlinear manifold learning methods, and underlying the main statistical tests used in the study, should be better discussed and justified.

You will also need to make some editorial changes so that it complies with our Guide to Authors at https://www.nature.com/natcomputsci/for-authors .

In particular, I would like to highlight the following points of our style:

Nature Computational Science titles should give a sense of the main new findings of a manuscript, and should not contain punctuation. Please keep in mind that we strongly discourage active verbs in titles, and that they should ideally fit within 150 characters each (including spaces).

Our papers are usually organized as follows: Introduction, Results, Discussion, and Methods. The Results section can contain a subsection that summarizes the methodology proposed in the paper, so that the results can be well understood, but detailed information about the methodology should be placed in the Methods section.

We encourage you to archive the data reported in your manuscript in an accessible, persistent repository. If your data are archived prior to the acceptance of your manuscript, please provide us with the full citation as soon as you receive it so that a link to the data can be included in the publication. See http://www.nature.com/authors/policies/availability.html for more information.

If your paper is accepted for publication, we will edit your display items electronically so they conform to our house style and will reproduce clearly in print. If necessary, we will re-size figures to fit single or double column width. If your figures contain several parts, the parts should form a neat rectangle when assembled. Choosing the right electronic format at this stage will speed up the processing of your paper and give the best possible results in print. If you are in doubt about the correct format for your figures after reading our guidelines, please ask the art editors for advice computationalscience@nature.com.

Figure legends must provide a brief description of the figure and the symbols used, including definitions of any error bars employed in the figures.

Please use the following link to submit your revised manuscript and a point-by-point response to the referees’ comments (which should be in a separate document to any cover letter):

[*REDACTED*]

To aid in the review process, we would appreciate it if you could also provide a copy of your manuscript files that indicates your revisions by making of use of Track Changes or similar mark-up tools. Please also ensure that all correspondence is marked with your Nature Computational Science reference number in the subject line.

In addition, please make sure to upload a Word Document or LaTeX version of your text, to assist us in the editorial stage.

To improve transparency in authorship, we request that all authors identified as ‘corresponding author’ on published papers create and link their Open Researcher and Contributor Identifier (ORCID) with their account on the Manuscript Tracking System (MTS), prior to acceptance. ORCID helps the scientific community achieve unambiguous attribution of all scholarly contributions. You can create and link your ORCID from the home page of the MTS by clicking on ‘Modify my Springer Nature account’. For more information please visit please visit www.springernature.com/orcid.

We hope to receive your revised paper within two weeks. If you cannot send it within this time, please let us know.

We look forward to hearing from you soon.

Best,
Fernando

--
Fernando Chirigati, PhD
Chief Editor, Nature Computational Science
Nature Portfolio

Reviewers comments:

Reviewer #1 (Remarks to the Author):

Thank you for this very interesting paper which makes use of newly available mobility data and ML methods to better understand the propagation of SARS-CoV-2 in the United States. My expertise lies in infectious disease epidemiology and the generation and use of mobility data so I will focus on those points directly.
- You mention some of these points in your discussion, but it would be useful to the reader to know a

bit more about the SafeGraph data generation process and how any biases built into it might affect your results. For example:
o Are these data or trends directly validated against any other sources?
o Could you replicate your results using data from Mapbox, Decartes Labs, Cuebiq, Facebook, Google, Apple or Camber Systems? While these providers don't have these data at the CBG level, they do often have them at the ZCTA or county level. Validation of both the trends in SafeGraph data and the robustness of your methods using other data sets would greatly support your conclusions.
o You note that app usership is opaque. This is a very important point and could use some investigation either in the discussion or in the supplement. From my understanding, contracts between SDK publishers and providers such as SafeGraph can vary from state to state and over time. The user base can also vary significantly between the very gradients that you use for evaluation. For example, if SafeGraph gets its data from Tinder, Waze and Weather Underground, the usage of these apps might vary greatly across demographic lines and even on an urban rural gradient. Is there any way to dig deeper and evaluate if signals that you identify are truly results of behavior change, or of the behavior that you happen to capture from the individual contributing data at a specific period of time? Incorporating data from other providers, or perhaps an aggregator such as Camber may be useful to forestall some of these questions.
o A key component of this relating to "staying at home" is how are people generating GPS traces if they stay at home? I imagine that it would depend on the app but the types of apps people use might change when they stay at home (I may not use Waze as much for example). Furthermore, if I'm working from home all day, I may not interact with my phone as much generating less data.
- On Page 3 while discussion the change in SafeGraph designation of home CBG, does this also affect the designation of number of people who stay at home?
- May be preference but you go into further description of SafeGraph metrics in the results on Page 11. They may be better placed in the data section of methods.
- How is home defined here? You say that it's the "location" where a user spends time at night but how big is this "location"? For example, Facebook uses 600m resolution tiles as their highest granularity. What does Safe Graph do and how might their choice of spatial buffer affect your analyses?
- What is the temporal aggregation of SafeGraph data? Is it done daily? If so do they adjust for each time zone or is it all from 0000-2400 UTC? If it is at UTC do you think that crossing time zones may cause issues in analysis? For example 0000 at UTC may be 1700 PST. Would dichotomizing mobility between days at 1700 cause issues in your analysis?
- For defining home location, what other cleaning or optimization steps are used by SafeGraph? Do they have a minimum number of interactions that a user must have per day for example? Do you know the average number of GPS traces that they have in their data and if this value varies across space and time?
- In other mobility datasets that I've seen this variation can go from a median of 20 GPS traces per day per user to less than 5 in rural regions affecting the signal that we were able to extract.
- I may have missed this but how well does the mapping of CBG to ZCTA work? Are there many CBGs which are on the border and would go to a different ZCTA if your specifications were changed?
- Trivial point but the maps could use a legend, even if it's just to say "Cluster A, Cluster B" etc.
- I've noted this quite a few times already and don't want to beat a dead horse here but could the

signals that you're getting simply be an artifact of the data generation process? Knowing more about the baseline data (# of traces per user, timeline dichotomization, # of publishers, # of apps, etc) or comparisons with other providers who may have similar data could help answer some of these questions. At the end of the day I don't think there is an obvious answer to this issue but a strong comparison with other dataset and sensitivity analyses based on your understanding of the data generation process can go a long way to strengthening your case.
- On page 8 you note that you use an alpha of 0.001. Why? Is this the standard for these methods or is there some adjustment for the type of testing that you're doing?
- Figure 3 could use a legend.
- Could you look into how your clusters track with NCHS categories of urbanicity? (https://www.cdc.gov/nchs/data_access/urban_rural.htm)
- The high mobility populations that you note on page 10 may also be producing data in very different ways. One of the things we've seen is that as people are able to stay home they use some services more (social media, games, news etc) and others less (outdoors apps, weather, navigation). Again the heterogeneity in app usage can drive a lot of differences between CBGs.
- Another concern of mine is the cleaning, smoothing and general optimization that providers may do in their processing. In many cases these are optimized for commercial purposes and may not provide direct information about general human behavior. For example, if an app cares more about travel to shopping areas it may not be capturing information when you're not moving at home.
- I really like Figure 5 and the clear message it sends in terms of the differentiation of your clusters. Again it would be interesting to look at this by NCHS categories while also digging deeper into how these different groups (% ages 18-29y for example) might generate different types of mobility data.
- Your results in Figure 7 are similar to ones I've seen when stratifying by urbanicity in the fact that rural areas seem to have more variability in general. It would be interesting to dig a bit deeper into that, and again, identify if there is a large change in the # of GPS traces per user / day across these categories.
- The points you make about income are interesting but I still wonder (not just for this paper but also for the ones that you've referenced) if this may simply be a measure of the types of apps these individuals use, the ability to stay at home and the need for essential travel. If I live in an urban area I may be able to have food and groceries delivered to my house. However, in rural areas I may need to drive to be able to cover my essential needs.
- Potential extra citation for the point you make on page 16 about the depopulation of urban areas: https://www.nature.com/articles/s41598-021-86297-w. As a note of transparency I contributed to this paper so please do not feel that you have to include the citation at all.
- On Page 17 you note that the clusters that stayed the home the most in the first few months had the lower cases per capita in following waves. I wonder if this may not simply be confounded by heterogeneity in masking and physical distancing measures.
- A key point of the paper is the potential utility of this method by decision makers. It would be useful to highlight this by describing the operational steps that can be taken by decision makes based on the results here.

Reviewer #2 (Remarks to the Author):

Levin et al use a variety of human movement data to better understand patterns of mobility during the COVID epidemic.

The authors use several new methods to describe new characteristics of the movement networks which is exciting. However, their link to epidemiological characteristics remained weak.

For example, the GMM clustering is interesting epidemiologically as you would expect more transmission to occur close to home? in the home? If so, could the authors attempt to model the specific relationship between their movement descriptors and the epidemiology of COVID19 in that place?

Even though the authors describe rough epidemiological characteristics in the section 4.5 I am left wondering about all the confounders? It remains difficult for the reader to really understand the biological importance of these findings.

I cannot comment on the robustness of the quantitative methods applied to the human mobility data.

Reviewer #3 (Remarks to the Author):

In this paper, the authors use mobility data (SafeGraph), Census data (ACS and TIGER/Line), and COVID-19 test and case data from Washington state to quantify the impact of human mobility (and relatedly non-pharmaceutical interventions designed to reduce mobility) on transmission of SARS-CoV-2. This is a novel paper that uses manifold learning to to cluster census block groups by their observed mobility patterns. The authors then evaluate the correlation between their clusters and socioeconomic variables as well as COVID-19 case/death counts for Washington state. The authors find that their clusters result in geographically connected regions, are correlated with income, and other socioeconomic measures, sudden changes in the number of people at risk in a given area, and COVID-19 cases.

Metholodigically, this paper is beyond my ability to thoroughly review. Instead, I focus on the substantive aread, framing, and clarity. I found each section of the paper to be well-written, but had difficulty connecting the sections in a logical pattern. However, the results are extensive and dense and the authors do a nice job of walking the reader through each result. Below, I list categorize my comments as major (should be considered before resubmission) and minor (clarificaiton will help the reader) in hopes that authors find them useful for creating a stronger manuscript.

Major

- It appears the objective of the paper is to use COVID-19 as a concrete example of how phone-based

mobility data, in combination with manifold learning, can be used to inform policy. It is, however, hard to discern if this is the case. The second to last sentence of the first paragraph in the introduction suggests it is the case but the rest of the introduction is less clear (e.g., "provide insight into behavioral differences", "reveal insights into epidemiologically relevant subpopulations", etc.). If these all fall under the broad category of informing policy, perhaps laying this out in the initial paragraph is helpful.
- It would be helpful to see a comparison of this novel method with a traditional or naive method. That mobility is highly correlated with area-level socioeconomic measures or are geographically clustered is not surprising given the US' deep history of segregation. Similarly, that mobility may provide some signal for COVID-19 cases (given both transmission but also the socioeconomic risk factors), is unsurprising. The paper argues this more complex method has value to add, and I want to believe it, but it is not clear how much more. I would like to see even very simple comparisons of correlation between each method and the ACS variables. Given a ranked list of COVID-19 cases, what is the correlation for (ranked) mobility vs the correlation for socioeconomic measures of interest? Not necessarily this exact example, but something to show the reader this is better by X amount.
- It is likely this framework could be applicable outside of COVID-19 and indeed given the widespread rollout of vaccines, it may strengthen the paper to use COVID-19 as a concrete example of a broader framework. For example, understanding human mobility during natural disasters, other pandemics, or seasonal changes would all still be very helpful for informing (non-COVID-19) policy.

Minor

- The plots are very pretty but it is not clear to me Figure 2i is necessary and should not be in the appendix with Figure S9 or S10. Instead, I wonder if it makes more sense to show 2ii, and 2iii, as well as color coded raw mobility for each cluster so we can see the within-cluster variation of mobility. (The example in Figure 1D should remain.)
- In the introduction, it's worth noting a fairly significant impact on morbidity (not just mortality) and it's unclear to me what the authors mean by increased "gender inequity" as Ref 3 does not seem to refer to gender inequity. Please remove or clarify and cite. Note that I would actually argue many more papers have pointed out substantial socioeconomic and racial/ethnic inequity and the authors should consider adding this in.
- The SVD section (3.1) and brief mentions of it in the introduction (Page 2) seem unnecessary and could be moved to the supplement (especially given how little they are mentioned in the results). I would suggest simply having an SVD section in the supplement, briefly mentioning that SVD is typical but inadequate and therefore you are using manifold learning. It's also not clear if the SVD-clustering method was also highly correlated with CT-variables? Was the only testing looking at the variation explained? Similar to comment above, it would be helpful for to see a simple comparison between methods.
- Similarly, I think Section 4.6 could be moved to the supplement to keep the results more streamlined.
- I believe SafeGraph data come from *smart*phones (i.e., not all *cell*phones) and that distinction should be made clearer since smartphones only cover about 70% of all cellphones in the US.

In summary, I believe the authors provide a clear application of a novel methods for using mobility data

to summarize highly complex human behavior. I would argue the authors sell themselves short with applications since this could be broadly applicable for a variety of health outcomes, natural disasters, etc. That said, the use of COVID-19 as a concrete example makes sense. I think the paper could be streamlined by removing some of the results (above) and the overall message can be distilled further.

Reviewer #4 (Remarks to the Author):

The authors describe a two-step data analysis for cell phone mobility data on the census-block-group geographic scale in the states California, Georgia, Texas, and Washington. The two steps involve using (comparatively new/less well known) manifold learning techniques for dimension reduction and (comparatively old/well known) Gaussian mixture models for cluster analysis in the space of reduced dimension.

The authors claim to find patterns of mobility behavior
* that align with stay-at-home orders,
* correlate with socioeconomic factors,
* cluster geographically,
* reveal subpopulations that likely migrated out of urban areas,
* and link to COVID-19 case counts.
They also claim that their analysis and approach provides policy makers a framework for interpreting mobility data (and behavior) to inform actions aimed at curbing the spread of COVID-19.

The combination of (a) unprecedented stay-at-home orders, (b) availability of cell phone mobility data, and (c) comparatively new nonlinear manifold learning methods presents a significant advancement in the application of manifold learning and clustering to real world data.
The authors show different behaviors - identified with their proposed methodology - for the early months of the epidemic are associated with cases later in the epidemic. This is a very important point, as it validates (or, at least, may allow to evaluate) certain policy decisions based on their long term effects, broken down to certain groups of people.

Since the focus is less on the conclusions and more on the methodology, I also focused my review on the details of the methodology. Before I go into details there, I want to address (a) some ethical concerns that are not addressed at all, and (b) one of the major claims of the authors: that their "analysis and approach provides policy makers a framework [...] to inform actions aimed at curbing the spread of COVID-19" (from the abstract).

Regarding ethics:
The authors use the database from SafeGraph, Inc. While I am relatively sure the company has some ethical guidelines and does a good job at anonymizing the data, the authors should have at least mentioned a few of the ethical complications that arise when tracking the positions of millions of

people, especially when cross-referencing this data with census information. Do all people involved know/sign something/get informed that these statistics are being computed all the time? Is it possible to opt-out? At least there should be a link to a specific website (probably at SafeGraph Inc.) where these issues are addressed. In the manuscript, it should be discussed if the analyses of mobility data from smartphones allow more severe intrusions in the live of people (what if a cell phone company sells a similar analysis to insurance companies? Etc.). These discussions do not need to be extensive, but at least a couple of sentences should adress the major issues.

Regarding informing policy decisions:
In my point of view, the state of the current work does not allow to inform policy decisions yet: the proposed framework involving dimension reduction and clustering - albeit interesting and an important step in the right direction - involves too many unknowns to be used directly to inform decisions. The additional analyses of the clusters, involving socioeconomic information and statistical tests, are what can ultimately inform the decisions. In their current state, the analysis of the found clusters mostly validates the dimension reduction and clustering methods by providing *plausible* clusters (i.e. clusters that can be rationalized through other means). It is not clear - as I also explain in more detail below - that the clustering method provides consistent and new results elsewhere (and also, importantly, for other pandemic-like situations apart from COVID-19).

Let me now focus my review on the combination of dimension reduction and clustering that is the basis of the later analyses by the authors.

In the abstract, the authors state that "Using manifold learning techniques, we find patterns of mobility behavior" - but the patterns are actually found using a Gaussian mixture model (GMM), the manifold learning techniques are only used for dimensionality reduction before applying the GMM. In contrast, for example a spectral clustering method would be much closer to "manifold learning for pattern detection", especially because it is closely related to Laplacian Eigenmaps and Diffusion Maps. I do not see a GMM as a manifold learning technique (it does not have a manifold assumption on the data), but the authors may convince me otherwise.

A much more important point (compared to the issue of terminology around "manifold learning") is that the authors do not discuss the assumptions underlying the linear and nonlinear manifold learning methods they employ. Instead they seem to have tested multiple methods and ultimately used the ones that "reduced the dimensionality of the data and identified a consistent tubular dense structure in the data" (quote from the supplement section 2). It is reasonable to approach the problem in this way, but with a framework to inform policy decisions in mind, and also for an analysis on this scale, checking the assumptions of the methods is crucial. The authors "found that Laplacian Eigenmaps, Locally Linear Embedding, and Isomaps" (again citing from section 2 in the supplement) worked best among the methods they tried, but these three methods are almost identical in what they assume about the data AND they also work almost identically, especially because the authors seem to be using the same distance metric between points (time series) - here, nearest neighbors with Euclidean distance in the ambient space.

The most crucial assumptions of the manifold learning methods above are (a) the data distribution is uniform (!) on the manifold in the ambient space, and (b) the manifold is compact, i.e. the density does not decay to zero toward any boundary. It is hard to argue that both assumptions are satisfied in this particular scenario. This leads me to believe that the alleged "robustness" of the results over different methods and parameters are (a) because the methods work almost the same and (b) the data itself is also extremely similar between states (all states considered are in the United States, all states were considered in the same time period, all data collection worked the same way, etc.).

There are remedies and updates to the methods that mitigate both the data density (e.g. using Diffusion Maps instead of Laplacian Eigenmaps) and the data density approaching zero (e.g. by using variable bandwidth kernels, or continuous nearest neighbor kernels, see
* doi: 10.3934/fods.2019001
* doi: 10.1016/j.acha.2015.03.002
). In my opinion, it is crucial to either update to these more recent methods, or discuss in detail why the assumptions for Laplacian Eigenmaps either (a) can be disregarded in this particular case, or (b) are satisfied (which I do not think they are). In particular: what would happen if the data was drawn uniformly over space, i.e. was not clustered toward cities? What would happen if GMM was applied to 14 SVD vectors (the same number as for LE), instead of 60? How do extreme outliers influence the results (e.g. faulty time series)? What happens if only half of the data is available, or twice as much (e.g. discussing convergence/ stability under more/less data)?

Regarding the statistical tests, I have less severe - but similar - concerns toward discussion of assumptions:

Two main statistical tests are used, Kolmogorov-Smirnov (to test if the speed to respond to stay-at-home orders in March 2020 is the same between identified clusters) and Jonckheere–Terpstra (to test if median stay-at-home fraction, population density, and household income of CBGs had a consistently decreasing trend for four of the five clusters).
I am not a statistician, so I cannot judge wether there are underlying, inherent flaws in using these tests for this particular data, but they seem appropriate for the tasks.
Nevertheless, the assumptions (and if/why they apply) of the two tests could be discussed in more detail, e.g. normality assumtion for the individual speed distributions for the Kolmogorov-Smirnov test. For the Jonckheere–Terpstra test, these assumptions may be harder to rationalize, see in particular Assumption 3 (independence of observations) and 4 (the distributions of the scores must be the same with the only possible difference being a shift in location) here:
https://statistics.laerd.com/spss-tutorials/jonckheere-terpstra-test-using-spss-statistics.php#assumptions

Related: figure 5 shows bar charts scaled to 100% that include data with N as small as 2 and 3. This may lead people to the wrong conclusions, because e.g. "same house, Georgia" and "same houes, Texas"

charts make it look like clusters B and C do not occur at the beginning, but this may just be because of missing data.

On a more subjective note, I feel that the paper will influence thinking in the field of cell phone mobility data analysis and be of interest to a wider computational science community of researchers; in particular for research with access to large volumes of time series data related to human activities (tracking devices for sport, cell phones, driving data from cars, etc).

I still think the work is highly relevant and important, and should eventually be published. The methodology the authors proposed - even with the flaws regarding their assumptions I outlined above - is still relatively recent and definitely an outstanding feature in the area of the analysis of mobility data. From an applied perspective, the authors show different behaviors identified with their methodology for the early months of the epidemic are associated with cases later in the epidemic, which may allow very important findings in the future.

Some technical issues:

-> regarding "Figure legend": Figure 4 describes the error bar in its caption, "Whiskers span the 95% range."
-> Figure 7 shows standard deviations (?) around mean curves, without clear explanation. What does "inter-quartile range" mean?

-> Abstract: "As COVID-19 cases resurge in the United States" will sound strange in 10 years (I hope...)
-> What about people that do not use smartphones? In the US that may be not an issue, but maybe in other countries. Do the authors just assume everybody has one, or are all conclusions "for the population of smartphone users"?
-> 4.3: "these four clusters come from the same distribution of values ($p<0.01$)" should list the exact p value
-> 4.4: "never-near-home" devices is a little strange; how do the authors know where the home of a device is if it is "never" home?
-> page 17: "affect the persistence of risk for coronavirus susceptibility and transmission" should replace "coronavirus" with SARS-COV-2 (unless the authors mean all types of coronaviruses, which would be strange)

Author Rebuttal to Initial comments

Dear Editor,

We thank you and the reviewers for the opportunity to submit a revision to our manuscript for review. We also thank the reviewers for the kind and encouraging comments about the work and manuscript: Reviewer 2 said the manuscript "uses several new methods to describe new characteristics of the movement networks which is exciting"; Reviewer 3 indicated the paper is "well-written" and the "authors do a nice job of walking the reader through each results"; and Reviewer 4 said the work could "influence thinking in the field of cell phone mobility data analysis and be of interest to a wider computational science community of researchers."

The comments and questions are fair and thorough; in addressing them, we believe the manuscript has been much improved. We have addressed yours and the reviewer comments in blue with modifications of the text of the manuscript and supplement in red, both in this response and in the text of the manuscript and supplement itself. We first directly respond to your high-level points; we then follow with more extensive reply to the individual referee's comments.

Thank you,

Joshua L. Proctor on behalf of all authors

- Please provide more details on the data generation process, as this is key to better understand the results obtained in the paper. Please also discuss the ethical implications that arise when collecting this type of data, since this can be useful to our readership.

We have addressed as many of the comments as possible with available data and methods from SafeGraph as well as some of the ethical implications for Reviewer 1 and 4. We have made substantial changes to the manuscript including modifications to the Methods. In the Discussion, we highlight the connection between data generation, ethical implications, and how those have downstream consequences relative to the limitations and challenges of our work.

- To better validate the robustness of the proposed methodology, and the obtained trends and results, it would be ideal to also experiment with other (similar) data, as suggested by Reviewer #1.

We thank Reviewer 1 for the suggestion. We addressed this comment in three ways. Firstly, we highlighted some of the recent literature that has investigated the consistency of metrics from different smart phone data aggregators, including the Weill et al. 2020 work. Secondly, we downloaded the publicly available Google and Facebook mobility data at the county level. Even with differing metrics and methodologies, a spot check on counties (additional figure included in the reviewer response) showed qualitatively similar behavior for SafeGraph data with Google and Facebook's data. Thirdly, we constructed a synthetic dataset for the github repository to test the methodology with a known "truth" data.

However, we were not able to find alternative data sources at the Census Block Group (CBG) geographic scale to reproduce our analyses; this scale is essential given our results show the heterogeneity within counties and that Cluster E is really a CBG geographic scale result, i.e, zooming in on CBGs around the University of Washington. Given our investigation here and the comments by the reviewers, we see future investigations expanding the scope across more states, counties, and data aggregators (SafeGraph, Facebook, Google, etc), especially since the methodology will efficiently scale.

- Please improve the link of the obtained results to epidemiological characteristics and their biological importance.

This is a great and subtle point. We have attempted to clarify this in the text of the manuscript. In particular, we believe mobility is a proxy for potential exposure to outside the household, but it could also be a reflection of demographic and socioeconomic factors for SARS-CoV-2 susceptibility and transmission. It's difficult to disentangle these with the unlinked datasets (as others in the community have mentioned). We believe the value here is to highlight the associations and show the *dynamic* link between mobility behaviors and COVID risk. We have made modifications to the Discussion around this point as well as indicating that it does not fully capture the risk.

- Comparison of the proposed method against a traditional or naive method is needed.

We have added an analysis using a naïve method. As expected and discussed in the manuscript, there are substantial similarities, especially in the bulk stay-at-home behavior. However, the naïve method does not identify the mobility behaviors around Cluster E; our framework picked up an "outlier" cluster E, which has distinct peaks in late June and late September, corresponding to activity at universities. We appreciate the comment and believe this helps strengthen our argument for a data-driven approach in comparing whole time-series versus pre-defined metrics. We added a new Supplemental Figure (S18, also shown in the reply to reviewers) and refer to it in the Discussion.

- Some reviewers argue that the framework, while interesting, might not be ready yet to inform policy decisions. Please better discuss this point in the paper, or change the story of the paper to better reflect what could be accomplished with the framework.

This was a great set of comments and suggestions. We agree. We have reframed the audience to be state or local epidemiologists. They would be able to integrate the insights from the framework we have developed here with the other data streams in order to provide recommendations to policy-makers. The epidemiologists are well-poised to understand the potential limitations in terms of data and methods and provide their recommendations with appropriate caveats. We have made changes to the Abstract, Introduction, and Discussion framing.

- The assumptions underlying the linear and nonlinear manifold learning methods, and underlying the main statistical tests used in the study, should be better discussed and justified.

We have investigated the underlying assumptions for both the manifold learning methods and the statistical tests. We have addressed Reviewer 4's comments about the statistical tests. At the suggestion of Reviewer 4, we tested a broader set of manifold learning methods by implementing Diffusion Maps with fixed and variable bandwidth kernels. We find that the results are quite robust, with some variability depending on the method parameters. We believe this helps address Reviewer 4's concerns about the data density and compactness of the mobility data distribution. We have expanded the supplement significantly to broaden these results and made modifications to the manuscript text.

You will also need to make some editorial changes so that it complies with our Guide to Authors at https://www.nature.com/natcomputsci/for-authors .

In particular, I would like to highlight the following points of our style:

Nature Computational Science titles should give a sense of the main new findings of a manuscript, and should not contain punctuation. Please keep in mind that we strongly discourage active verbs in titles, and that they should ideally fit within 150 characters each (including spaces).

Thank you for pointing this out. We have changed the title to the following to remove the punctuation.
Insights into population behavior during the COVID-19 pandemic from cell phone mobility data and manifold learning

Our papers are usually organized as follows: Introduction, Results, Discussion, and Methods. The Results section can contain a subsection that summarizes the methodology proposed in the paper, so that the results can be well understood, but detailed information about the methodology should be placed in the Methods section.

We have moved some material from "Results" to "Methods". We combined our "Data" and "Methods" sections and moved it to the end.

We encourage you to archive the data reported in your manuscript in an accessible, persistent repository. If your data are archived prior to the acceptance of your manuscript, please provide us with the full citation as soon as you receive it so that a link to the data can be included in the publication. See http://www.nature.com/authors/policies/availability.html for more information.

Thank you. We are not able to archive the data directly, but we report how to access it via SafeGraph. We have constructed a github repository for all code. In addition, we have created a synthetic dataset to help validate the method, but also enable new users of the code-base.

If your paper is accepted for publication, we will edit your display items electronically so they conform to our house style and will reproduce clearly in print. If necessary, we will re-size figures to fit single or double column width. If your figures contain several parts, the parts should form a neat rectangle when

assembled. Choosing the right electronic format at this stage will speed up the processing of your paper and give the best possible results in print. If you are in doubt about the correct format for your figures after reading our guidelines, please ask the art editors for advice computationalscience@nature.com. Figure legends must provide a brief description of the figure and the symbols used, including definitions of any error bars employed in the figures.

Please use the following link to submit your revised manuscript and a point-by-point response to the referees' comments (which should be in a separate document to any cover letter):

** This url links to your confidential homepage and associated information about manuscripts you may have submitted or be reviewing for us. If you wish to forward this e-mail to co-authors, please delete this link to your homepage first. **

To aid in the review process, we would appreciate it if you could also provide a copy of your manuscript files that indicates your revisions by making of use of Track Changes or similar mark-up tools. Please also ensure that all correspondence is marked with your Nature Computational Science reference number in the subject line.

In addition, please make sure to upload a Word Document or LaTeX version of your text, to assist us in the editorial stage.

To improve transparency in authorship, we request that all authors identified as 'corresponding author' on published papers create and link their Open Researcher and Contributor Identifier (ORCID) with their account on the Manuscript Tracking System (MTS), prior to acceptance. ORCID helps the scientific community achieve unambiguous attribution of all scholarly contributions. You can create and link your ORCID from the home page of the MTS by clicking on 'Modify my Springer Nature account'. For more information please visit please visit www.springernature.com/orcid.

We hope to receive your revised paper within two weeks. If you cannot send it within this time, please let us know.

Response to referee comments:

We thank all of the reviewers for their kind and constructive comments, questions, and suggestions. We believe the manuscript is much improved. Our replies to your reviewers are below in blue with manuscript and supplement modifications in red.

Reviewer #1 (Remarks to the Author):

Thank you for this very interesting paper which makes use of newly available mobility data and ML methods to better understand the propagation of SARS-CoV-2 in the United States. My expertise lies in infectious disease epidemiology and the generation and use of mobility data so I will focus on those points directly.

We thank the reviewer for the kind words about the paper and appreciate the comments and questions.

- You mention some of these points in your discussion, but it would be useful to the reader to know a bit more about the SafeGraph data generation process and how any biases built into it might affect your results. For example:

- Are these data or trends directly validated against any other sources?
- Could you replicate your results using data from Mapbox, Decartes Labs, Cuebiq, Facebook, Google, Apple or Camber Systems? While these providers don't have these data at the CBG level, they do often have them at the ZCTA or county level. Validation of both the trends in SafeGraph data and the robustness of your methods using other data sets would greatly support your conclusions.

Your two first bullet points are related; here, we address both. This is a great set of questions. Weill et al. 2020 showed the general trends from March to May in 2020 were qualitatively similar across SafeGraph, PlaceIQ, and Google data. We have added a sentence in the discussion to directly address this concern with this citation.

"However, the trends of similar mobility metrics from different sources SafeGraph, PlaceIQ, and Google data are qualitatively similar [8]."

We also downloaded the Google and Facebook publicly accessible mobility data to examine this directly based on your comment. We realize the metrics across SafeGraph, Google, and Facebook data are related, but different. Here, we spot check Weill et al.'s observation, for one of our focus regions: Washington state. Here are the trends for King County and Spokane County:

[REDACTED]

We see that the trends are qualitatively similar, matching previous observations. We believe Weill et al. 2020 and this comparison provides evidence that SafeGraph data is consistent with major available mobility data sources.

With regards to applying this method to other datasets such as these, we were not able to find the data at the CBG level, only at the county level (as you mentioned). In the results of our analysis, we found

CBGs often having different trends within the same county. In addition, one of the key epidemiological results of the paper around the purple cluster is smoothed over at the county level. Unfortunately, it's unclear how to directly validate our method and results on a dataset at the county level. We did create a synthetic dataset tied to Washington state CBG scale to validate the methodology. The dataset is in the code repository and shows how the method can group similar time-series. Lastly, we appreciate the suggestion and see the potential for future work where we use these methods at the county level, but across the whole country and across multiple datasets. We have added a sentence in the last paragraph of this discussion along these lines:

"In future work, we see leveraging this framework to integrate similar data sources, such as Facebook and Google's mobility data, across a much wider geographic scope to minimize the bias of any one source."

- You note that app usership is opaque. This is a very important point and could use some investigation either in the discussion or in the supplement. From my understanding, contracts between SDK publishers and providers such as SafeGraph can vary from state to state and over time. The user base can also vary significantly between the very gradients that you use for evaluation. For example, if SafeGraph gets its data from Tinder, Waze and Weather Underground, the usage of these apps might vary greatly across demographic lines and even on an urban rural gradient. Is there any way to dig deeper and evaluate if signals that you identify are truly results of behavior change, or of the behavior that you happen to capture from the individual contributing data at a specific period of time? Incorporating data from other providers, or perhaps an aggregator such as Camber may be useful to forestall some of these questions.

We agree this is an important point. Unfortunately, we have investigated the SafeGraph documentation and did not find any specific information that could fully address this question. As we discussed in the response for the last question, we did look across publishers to see the trends are qualitatively similar at the county level between Spokane and King County; these sources likely have somewhat different user and application bases. In addition, the counties themselves are quite different in terms of political leanings, demographics, and socioeconomic groups. We acknowledge that this still does not remove the possibility that each of the sources are biased in the same way. We have made sure to highlight this concern in the discussion:

Different states and segments of the population may have different levels of coverage, including over the course of the pandemic, that are hard to correct for [40].

- A key component of this relating to "staying at home" is how are people generating GPS traces if they stay at home? I imagine that it would depend on the app but the types of apps people use might change when they stay at home (I may not use Waze as much for example). Furthermore,

if I'm working from home all day, I may not interact with my phone as much generating less data.

SafeGraph observed that the number of tracked devices did seem to drop early in the pandemic, possibly because people were not using their phones. We also expect that specific app usage changed during the pandemic as well. This likely affected the denominator of the number of tracked devices. The SafeGraph estimate of the number of people who stayed home is probably incorrect, but the relative percentage and trend is likely robust. Similar to the last comment, we believe the following statement describes this important limitation:

"Different states and segments of the population may have different levels of coverage, including over the course of the pandemic, that are hard to correct for [40]."

- On Page 3 while discussion the change in SafeGraph designation of home CBG, does this also affect the designation of number of people who stay at home?

We were unable to detect obvious jumps in the number of devices tracked or the number of devices staying at home on the date of the algorithm change, besides those in "cluster E" as described in Results. We suspect that for the other clusters, the impact could have been more gradual because of the rolling window calculation.

- May be preference but you go into further description of SafeGraph metrics in the results on Page 11. They may be better placed in the data section of methods.

Thank you for the suggestion. We have moved some of this technical information from the Results to Methods.

- How is home defined here? You say that it's the "location" where a user spends time at night but how big is this "location"? For example, Facebook uses 600m resolution tiles as their highest granularity. What does Safe Graph do and how might their choice of spatial buffer affect your analyses?

Home is defined at the Geohash 7 level (153x153m). We have added a sentence to Section 4.1:

SafeGraph defines a person's "home" to be the location where the mobile device is detected most at night (from 6pm to 7am) over a 6-week period [27]. Location is defined at the Geohash-7 level (about 153 meters by 153 meters). If a person spends enough time in a new location, that new location can become the device's "home".

- What is the temporal aggregation of SafeGraph data? Is it done daily? If so do they adjust for each time zone or is it all from 0000-2400 UTC? If it is at UTC do you think that crossing time zones may cause

issues in analysis? For example 0000 at UTC may be 1700 PST. Would dichotomizing mobility between days at 1700 cause issues in your analysis?

Local time is used in their home assignments. (Reference [41], at https://docs.safegraph.com/docs/places-manual#section-safe-graph-common-nighttime-location-algorithm)

- For defining home location, what other cleaning or optimization steps are used by SafeGraph? Do they have a minimum number of interactions that a user must have per day for example? Do you know the average number of GPS traces that they have in their data and if this value varies across space and time?

These details are not available from SafeGraph.

- In other mobility datasets that I've seen this variation can go from a median of 20 GPS traces per day per user to less than 5 in rural regions affecting the signal that we were able to extract.

Unfortunately, individual GPS traces are not available or discussed in SafeGraph.

- I may have missed this but how well does the mapping of CBG to ZCTA work? Are there many CBGs which are on the border and would go to a different ZCTA if your specifications were changed?

The mapping from CBGs to ZCTAs is briefly described in Methods under "Census and geographic data". The mapping from CBGs to ZCTAs is imperfect. The CBG is assigned to the ZCTA that contains the largest share of its area. This assumption generally works well in urban areas, where CBGs are relatively small, but works less well in rural areas where CBGs can be nearly as large as ZCTAs. The CBGs that fall on the border of two ZCTAs could be assigned to different ZCTA under different rules. We had also tried assigning each CBG to multiple ZCTAs based on degree of overlap, but this turned out to be more complicated and produced artifacts near bodies of water and in rural areas. For example, the area of a CBG on the waterfront might be mostly over the water, but we know that people actually live on the land, which caused some CBGs to be assigned to non-obvious ZCTAs.

- Trivial point but the maps could use a legend, even if it's just to say "Cluster A, Cluster B" etc.

Thanks for pointing this out. We have added legends to the maps.

- I've noted this quite a few times already and don't want to beat a dead horse here but could the signals that you're getting simply be an artifact of the data generation process? Knowing more about the baseline data (# of traces per user, timeline dichotomization, # of publishers, # of apps, etc) or comparisons with other providers who may have similar data could help answer some of these questions. At the end of the day I don't think there is an obvious answer to this issue but a strong

comparison with other dataset and sensitivity analyses based on your understanding of the data generation process can go a long way to strengthening your case.

We appreciate the comment and share the same concern. We hope that our earlier discussions have helped strengthen our case, but also provided the appropriate descriptions about the limitations based on data privacy.

- On page 8 you note that you use an alpha of 0.001. Why? Is this the standard for these methods or is there some adjustment for the type of testing that you're doing?

We have replaced this with a more standard reporting of the significance level of ($p<0.01$).

- Figure 3 could use a legend.

We have added a legend.

- Could you look into how your clusters track with NCHS categories of urbanicity? (https://www.cdc.gov/nchs/data_access/urban_rural.htm)

We added a new supplemental Figure (S16) that shows the proportion of CBGs in each cluster by NCHS urbanization category – we show one panel of this new figure below. The NCHS categorizes counties by urbanicity, not CBGs, so all CBGs within a county will be assigned the same category. We found trends consistent with the population density boxplot in Figure 4, where high density (urban/suburban) populations stayed home the most and low density (or rural) populations stayed home the least.

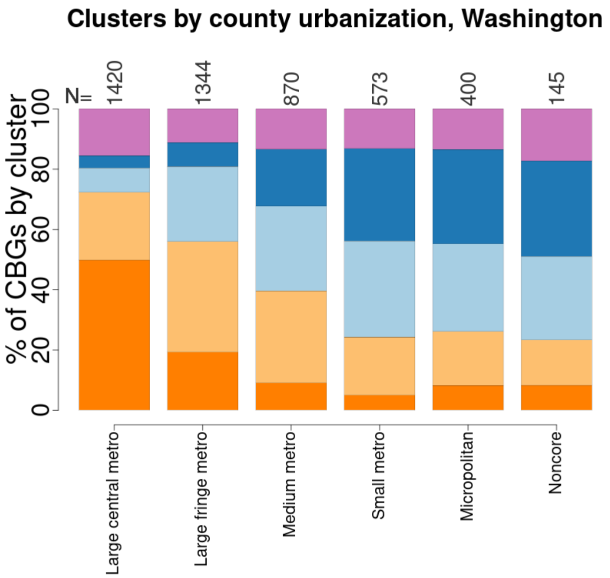

- The high mobility populations that you note on page 10 may also be producing data in very different ways. One of the things we've seen is that as people are able to stay home they use some services more (social media, games, news etc) and others less (outdoors apps, weather, navigation). Again the heterogeneity in app usage can drive a lot of differences between CBGs.

As mentioned earlier, we have added a sentence to the Discussion acknowledging that the opacity of the data collection could introduce biases. For your specific comment about the high mobility populations, we acknowledge the possibility, but also note that the high mobility clusters had a high proportion of phones that were only detected away from their previously inferred "homes". We believe this suggests that these people are regularly using smartphones and are mobile.

- Another concern of mine is the cleaning, smoothing and general optimization that providers may do in their processing. In many cases these are optimized for commercial purposes and may not provide direct information about general human behavior. For example, if an app cares more about travel to shopping areas it may not be capturing information when you're not moving at home.

This is a good point too. Unfortunately, we do not have access to this specific information, but we do find comfort in the earlier comparisons with other mobility metrics from other providers.

- I really like Figure 5 and the clear message it sends in terms of the differentiation of your clusters. Again it would be interesting to look at this by NCHS categories while also digging deeper into how these different groups (% ages 18-29y for example) might generate different types of mobility data.

Unfortunately, the data is aggregated at the CBG level, so we can observe higher mobility in areas with a high proportion of 18-29y, but we can't see what the actual young adults in those areas are doing. In the discussion, we do note that some of the areas in King County are associated with neighborhoods near the University of Washington, South Lake Union, and Capitol Hill which consists of students from the university and young professionals near the Seattle Amazon headquarters. However, we cannot directly link these mobility behaviors to those individuals; similarly, it is too speculative for this paper, but there was a specific UW COVID outbreak in the Greek system of UW from the fall of 2020 which (in terms of CBGs) is partially contained in the purple mobility cluster.

- Your results in Figure 7 are similar to ones I've seen when stratifying by urbanicity in the fact that rural areas seem to have more variability in general. It would be interesting to dig a bit deeper into that, and again, identify if there is a large change in the # of GPS traces per user / day across these categories.

We added a plot of the number of devices detected each day in Washington state by cluster (Figure S20) and refer to it in the Data section. We did not observe obvious trends in the number of mobile devices tracked.

- The points you make about income are interesting but I still wonder (not just for this paper but also for the ones that you've referenced) if this may simply be a measure of the types of apps these individuals use, the ability to stay at home and the need for essential travel. If I live in an urban area I may be able to have food and groceries delivered to my house. However, in rural areas I may need to drive to be able to cover my essential needs.

It is difficult to disentangle the relationships between income, rurality, and mobility. It seems plausible that wealthy urban-dwellers are more likely to work remotely and have groceries delivered, while these options are not available at any income level in some rural areas. However, it's unclear whether this difference in types of apps used would change the mobility trends we are observing. As a scientific community, it would be helpful to understand the consistency across groups and geographic regions. We have added this sentence to the manuscript:

Leveraging cell-phone mobility data to understand the COVID-19 pandemic has been widely discussed, including the challenge of understanding any potential ascertainment bias [Grantz et al]; similar to those discussions, if data providers offered insight into these potential biases, then the translation of works such as ours to policy-makers would be more strongly enabled.

- Potential extra citation for the point you make on page 16 about the depopulation of urban areas: https://www.nature.com/articles/s41598-021-86297-w. As a note of transparency I contributed to this paper so please do not feel that you have to include the citation at all.

Thank you for the reference. We have added this citation – some of our colleagues moved to more rural locations for telework so we are glad to acknowledge that movement was not spurred only by job losses.

- On Page 17 you note that the clusters that stayed the home the most in the first few months had the lower cases per capita in following waves. I wonder if this may not simply be confounded by heterogeneity in masking and physical distancing measures.

This a great point. However, it's proven a challenge to get masking and physical distancing data at this geographic and temporal resolution. We have anecdotal evidence about adherence to NPIs in the areas that we live as compared to areas in the same state, especially over the course of the pandemic. A somewhat simpler explanation, though, is that mobility characteristics of areas and groups, such as wealthier workers in technology that can stay home more, are less exposed to COVID.

- A key point of the paper is the potential utility of this method by decision makers. It would be useful to highlight this by describing the operational steps that can be taken by decision makes based on the results here.

Thank you for the comment. We do think that this method and type of data will be useful by decision-makers. Given comments by you and other reviewers, we have decided to modify the framing of the utility to decision-makers as more downstream. We believe that state epidemiologists could leverage this tool to integrate these mobility insights, with the caveats about potential data biases, with other pandemic surveillance to distill information into recommendations for policy-makers to inform intervention allocation efforts. We have addressed this by striking out a few sentences in the introduction and discussion, but adding the following in the concluding paragraph:

Abstract:

The analysis and approach provides \st{policy makers} \edit{local epidemiologists} a framework for interpreting mobility data and behavior to inform \edit{policy makers'} decision-making aimed at curbing the spread of COVID-19.

Introduction:

We believe the approach and insights in the work could be leveraged by local epidemiologists and integrated with other surveillance indicators to provide local public health officials a holistic recommendation as they decide on interventions such as educational campaigns by geographic area and socioeconomic status.

Discussion:

State and local epidemiologists can use this tool to integrate mobility insights with other pandemic surveillance indicators to help assess the impacts of policy by geographic regions and distill these data to provide further recommendations to policy-makers.

Reviewer #2 (Remarks to the Author):

Levin et al use a variety of human movement data to better understand patterns of mobility during the COVID epidemic.

The authors use several new methods to describe new characteristics of the movement networks which is exciting. However, their link to epidemiological characteristics remained weak.

For example, the GMM clustering is interesting epidemiologically as you would expect more transmission to occur close to home? in the home? If so, could the authors attempt to model the specific relationship between their movement descriptors and the epidemiology of COVID19 in that place?

The GMM identifies populations at the CBG geographic resolution that have similar stay-at-home time-series in a lower dimensional space; it does not, though, take into account the geographic relationship between CBGs. We are not suggesting that transmission necessarily occurs closer to home or in the home, but rather that different mobility behaviors (at the population scale) could lead to differential risks of COVID-19 in that population. We cannot directly link individual level movement and risk of COVID-19, since the datasets are not themselves linked. We have, however, attempted to show the association between our mobility clusters with COVID-19 case count in the CBG. Moreover, we find that the mobility clusters are also associated with future COVID-19 case burden.

Even though the authors describe rough epidemiological characteristics in the section 4.5 I am left wondering about all the confounders? It remains difficult for the reader to really understand the biological importance of these findings.

We agree that there are many confounders, such as income, wealth, and occupation. In order to highlight this, we have added a statement to the Discussion acknowledging that although mobility is associated with higher COVID case rates, mobility is associated with socioeconomic factors that also contribute to COVID risk.

"It is likely that mobility contributes to but does not fully capture COVID risk."

This statement now precedes the following sentence:

"Mobility may be a proxy for potential exposure to outside the household, but also reflect demographic and socioeconomic factors that affect the persistence of risk for SARS-CoV-2 susceptibility and transmission."

We hope this helps improve the narrative clarity about the implications of our work where we have focused on the links between behavior and transmission risk, but also acknowledging the limitations due to the complex link between behavior, socioeconomic factors, and others.

I cannot comment on the robustness of the quantitative methods applied to the human mobility data.

Reviewer #3 (Remarks to the Author):

In this paper, the authors use mobility data (SafeGraph), Census data (ACS and TIGER/Line), and COVID-19 test and case data from Washington state to quantify the impact of human mobility (and relatedly non-pharmaceutical interventions designed to reduce mobility) on transmission of SARS-CoV-2. This is a novel paper that uses manifold learning to to cluster census block groups by their observed mobility patterns. The authors then evaluate the correlation between their clusters and socioeconomic variables as well as COVID-19 case/death counts for Washington state. The authors find that their clusters result in geographically connected regions, are correlated with income, and other socioeconomic measures, sudden changes in the number of people at risk in a given area, and COVID-19 cases.

Metholodigically, this paper is beyond my ability to thoroughly review. Instead, I focus on the substantive aread, framing, and clarity. I found each section of the paper to be well-written, but had difficulty connecting the sections in a logical pattern. However, the results are extensive and dense and the authors do a nice job of walking the reader through each result. Below, I list categorize my comments as major (should be considered before resubmission) and minor (clarificaiton will help the reader) in hopes that authors find them useful for creating a stronger manuscript.

We thank the reviewer for the kind comment about the writing. We have attempted to address the issue around connecting the sections in a logical pattern. Specifically, we have modified the outline paragraph (last) in the Introduction to provide more clarity on the narrative link across results subsections.

Major

- It appears the objective of the paper is to use COVID-19 as a concrete example of how phone-based mobility data, in combination with manifold learning, can be used to inform policy. It is, however, hard to discern if this is the case. The second to last sentence of the first paragraph in the introduction suggests it is the case but the rest of the introduction is less clear (e.g., "provide insight into behavioral

differences", "reveal insights into epidemiologically relevant subpopulations", etc.). If these all fall under the broad category of informing policy, perhaps laying this out in the initial paragraph is helpful.

Thank you for the great comment. We do believe this work shows a real example of how phone-based mobility data can be analyzed to help inform policy. However, we have decided to reframe this connection based on yours and other reviewer's comments. Given the limitations of the current mobility data aggregations, we think the current audience is for state and local epidemologists. We believe the epidemiologists would be in the best position to integrate these insights with other surveillance indicators, including the assessment of the impact of NPIs by geographic areas, and provide recommendations to policy makers. We have changed the Abstract, Introduction, and Discussion to reflect this.

Abstract:

"The analysis and approach provides \st{policy makers} \edit{local epidemiologists} a framework for interpreting mobility data and behavior to inform \edit{policy makers'} decision-making aimed at curbing the spread of COVID-19."

Introduction:

"We believe the approach and insights in the work could be leveraged by local epidemiologists and integrated with other surveillance indicators to provide local public health officials a holistic recommendation as they decide on interventions such as educational campaigns by geographic area and socioeconomic status."

Discussion:

"State and local epidemiologists can use this tool to integrate mobility insights with other pandemic surveillance indicators to help assess the impacts of policy by geographic regions and distill these data to provide further recommendations to policy-makers."

- It would be helpful to see a comparison of this novel method with a traditional or naive method. That mobility is highly correlated with area-level socioeconomic measures or are geographically clustered is not surprising given the US' deep history of segregation. Similarly, that mobility may provide some signal for COVID-19 cases (given both transmission but also the socioeconomic risk factors), is unsurprising. The paper argues this more complex method has value to add, and I want to believe it, but it is not clear how much more. I would like to see even very simple comparisons of correlation between each method and the ACS variables. Given a ranked list of COVID-19 cases, what is the correlation for (ranked) mobility vs the correlation for socioeconomic measures of interest? Not necessarily this exact example, but something to show the reader this is better by X amount.

Thank you for the comments and suggestions. We have added a new Supplemental Figure (S19, shown below) and refer to it in the Discussion. One naïve approach is to classify zip codes by the average % of people who stay home over a time window. The left panel is a plot of the per capita number of COVID-19 cases in Washington State by “stay-at-home" quartile, where zip codes in quartile 1 stayed home the least and quartile 4 stayed home the most. It looks similar to the cases for clusters A-D (right panel) from the manuscript.

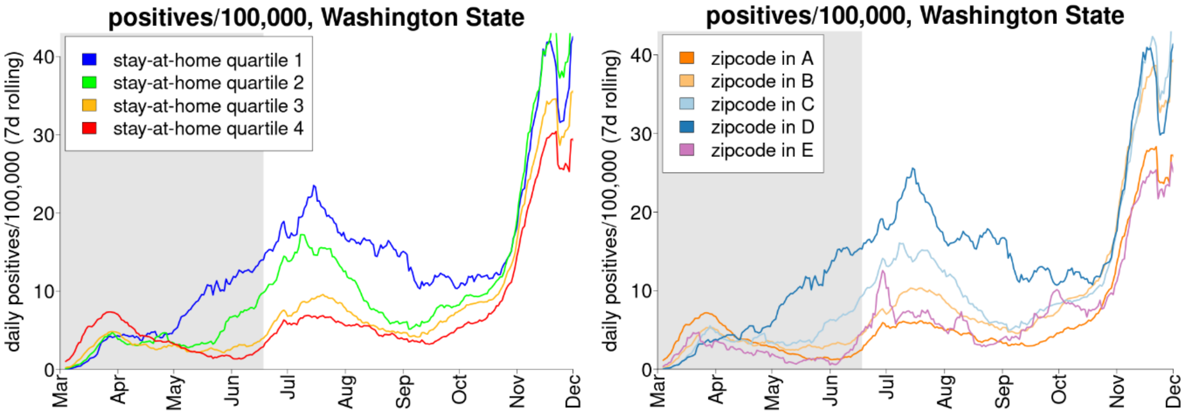

The key difference between these two, though, is that our method picked up an “outlier” cluster E, which has distinct peaks in late June and late September (this corresponded to activities at universities). We believe this cluster E is one piece of evidence for the value of using an approach like this; we do not pre-identify a metric, instead the methodology looks for similarities and differences with the time-series. We added a statement to the Discussion that our approach revealed the population that seemed to migrate at the beginning of the pandemic due to a quirk in the SafeGraph data. This would have been hard to predict a priori if we had just used SES covariates from the Census.

Text addition:

“If we had clustered CBGs by average behavior over time, we would still have found that the number of cases was highest among those who stayed home the least, but we would have completely missed the population that migrated early in the pandemic and had distinct outbreaks (Figure S18).”

With regards to the added value aspect of your question, we see identifying Cluster E as important since it would not have been clear how to investigate the metrics or associations you suggest. In addition, we find the fact this cluster has distinct epidemiological dynamics (outbreaks) and it’s strongly associated with renters, young professionals, students, etc really provided insights that would have been difficult to identify and group a priori.

- It is likely this framework could be applicable outside of COVID-19 and indeed given the widespread rollout of vaccines, it may strengthen the paper to use COVID-19 as a concrete example of a broader framework. For example, understanding human mobility during natural disasters, other pandemics, or seasonal changes would all still be very helpful for informing (non-COVID-19) policy.

Thank you for this comment; it's also been mentioned by another reviewer. We have added a prospective looking statement in the Discussion to highlight this research direction and opportunity:

"In addition, our framework could be useful beyond the current COVID-19 pandemic where understanding human mobility and behavior would help optimize interventions such as natural disasters, seasonal movements, or even a new pandemic."

Minor

- The plots are very pretty but it is not clear to me Figure 2i is necessary and should not be in the appendix with Figure S9 or S10. Instead, I wonder if it makes more sense to show 2ii, and 2iii, as well as color coded raw mobility for each cluster so we can see the within-cluster variation of mobility. (The example in Figure 1D should remain.)

We appreciate the comment. However, we believe showing how the data lies in these embedding spaces is a key message of the main manuscript; Figure 2i helps illustrate that this is qualitatively similar across multiple states. In Supplement Figure S20, we have an illustration of the within-cluster and between-cluster variation of mobility.

- In the introduction, it's worth noting a fairly significant impact on morbidity (not just mortality) and it's unclear to me what the authors mean by increased "gender inequity" as Ref 3 does not seem to refer to gender inequity. Please remove or clarify and cite. Note that I would actually argue many more papers have pointed out substantial socioeconomic and racial/ethnic inequity and the authors should consider adding this in.

Thank you for pointing this out. We have edited the sentence and added citations to add morbidity and focus on socioeconomic inequity.

"The ongoing COVID-19 pandemic has had a devastating impact on mortality [1], morbidity [2], and economic activity [3] leading to increased food insecurity, poverty, and socioeconomic inequity [2,4,5]."

- The SVD section (3.1) and brief mentions of it in the introduction (Page 2) seem unnecessary and could be moved to the supplement (especially given how little they are mentioned in the results). I would

suggest simply having an SVD section in the supplement, briefly mentioning that SVD is typical but inadequate and therefore you are using manifold learning. It's also not clear if the SVD-clustering method was also highly correlated with CT-variables? Was the only testing looking at the variation explained? Similar to comment above, it would be helpful for to see a simple comparison between methods.

Thank you for the suggestion. Since we only briefly mention the SVD results, we have moved the SVD method section to the Supplement. The SVD and clustering is indeed inadequate and does not provide a robust set of cluster memberships; we discuss this result in more detail in the first Supplement section. We do, however, think the narrative linking the rich history of dynamical systems, SVD, and the recent transition to manifold learning is valuable to contextualize this approach and link to ongoing research efforts in applied mathematics.

- Similarly, I think Section 4.6 could be moved to the supplement to keep the results more streamlined.

We agree – we removed Section 4.6 and now reference the supplement a bit earlier in the Results (now Section 2.1).

- I believe SafeGraph data come from *smart*phones (i.e., not all *cell*phones) and that distinction should be made clearer since smartphones only cover about 70% of all cellphones in the US.

Good point. We have changed the text to "smartphone" where needed.

In summary, I believe the authors provide a clear application of a novel methods for using mobility data to summarize highly complex human behavior. I would argue the authors sell themselves short with applications since this could be broadly applicable for a variety of health outcomes, natural disasters, etc. That said, the use of COVID-19 as a concrete example makes sense. I think the paper could be streamlined by removing some of the results (above) and the overall message can be distilled further.

Reviewer #4 (Remarks to the Author):

The authors describe a two-step data analysis for cell phone mobility data on the census-block-group geographic scale in the states California, Georgia, Texas, and Washington. The two steps involve using (comparatively new/less well known) manifold learning techniques for dimension reduction and (comparatively old/well known) Gaussian mixture models for cluster analysis in the space of reduced dimension.

The authors claim to find patterns of mobility behavior

* that align with stay-at-home orders,
 * correlate with socioeconomic factors,
 * cluster geographically,
 * reveal subpopulations that likely migrated out of urban areas,
 * and link to COVID-19 case counts.

They also claim that their analysis and approach provides policy makers a framework for interpreting mobility data (and behavior) to inform actions aimed at curbing the spread of COVID-19.

The combination of (a) unprecedented stay-at-home orders, (b) availability of cell phone mobility data, and (c) comparatively new nonlinear manifold learning methods presents a significant advancement in the application of manifold learning and clustering to real world data.

The authors show different behaviors - identified with their proposed methodology - for the early months of the epidemic are associated with cases later in the epidemic. This is a very important point, as it validates (or, at least, may allow to evaluate) certain policy decisions based on their long term effects, broken down to certain groups of people.

Since the focus is less on the conclusions and more on the methodology, I also focused my review on the details of the methodology. Before I go into details there, I want to address (a) some ethical concerns that are not addressed at all, and (b) one of the major claims of the authors: that their "analysis and approach provides policy makers a framework [...] to inform actions aimed at curbing the spread of COVID-19" (from the abstract).

Regarding ethics:

The authors use the database from SafeGraph, Inc. While I am relatively sure the company has some ethical guidelines and does a good job at anonymizing the data, the authors should have at least mentioned a few of the ethical complications that arise when tracking the positions of millions of people, especially when cross-referencing this data with census information. Do all people involved know/sign something/get informed that these statistics are being computed all the time? Is it possible to opt-out? At least there should be a link to a specific website (probably at SafeGraph Inc.) where these issues are addressed. In the manuscript, it should be discussed if the analyses of mobility data from smartphones allow more severe intrusions in the live of people (what if a cell phone company sells a similar analysis to insurance companies? Etc.). These discussions do not need to be extensive, but at least a couple of sentences should address the major issues.

Thank you for this important comment regarding ethics. We now cite the SafeGraph privacy policy page and have added sentences around ethical implications and opting out in the Discussion with the implications around data limitations for our framework. Unfortunately, it’s not clear from our reading of the privacy policy page what more severe intrusions in the lives of people are possible.

Regarding informing policy decisions:
In my point of view, the state of the current work does not allow to inform policy decisions yet: the proposed framework involving dimension reduction and clustering - albeit interesting and an important step in the right direction - involves too many unknowns to be used directly to inform decisions. The additional analyses of the clusters, involving socioeconomic information and statistical tests, are what can ultimately inform the decisions. In their current state, the analysis of the found clusters mostly validates the dimension reduction and clustering methods by providing *plausible* clusters (i.e. clusters that can be rationalized through other means). It is not clear - as I also explain in more detail below - that the clustering method provides consistent and new results elsewhere (and also, importantly, for other pandemic-like situations apart from COVID-19).

We thank you for the assessment. We have re-framed the narrative and implications given yours and other's comments. Instead of focusing on directly informing policy makers, we have instead focused on local and state epidemiologists. In addition, we have framed this as a complementary tool to other surveillance data and indicators where there needs to be an awareness of the challenges and limitations from both the data (see Reviewer 1 comments) as well as on the method side; we believe epidemiologists would be the best to integrate these insights with other data to provide recommendations to policy makers. We have modified sentences in the Abstract, Introduction, and Discussion accordingly.

Abstract:

"The analysis and approach provides \st{policy makers} \edit{local epidemiologists} a framework for interpreting mobility data and behavior to inform \edit{policy makers'} decision-making aimed at curbing the spread of COVID-19."

Introduction:

"We believe the approach and insights in the work could be leveraged by local epidemiologists and integrated with other surveillance indicators to provide local public health officials a holistic recommendation as they decide on interventions such as educational campaigns by geographic area and socioeconomic status."

Discussion:

"State and local epidemiologists can use this tool to integrate mobility insights with other pandemic surveillance indicators to help assess the impacts of policy by geographic regions and distill these data to provide further recommendations to policy-makers."

Let me now focus my review on the combination of dimension reduction and clustering that is the basis

of the later analyses by the authors.

In the abstract, the authors state that "Using manifold learning techniques, we find patterns of mobility behavior" - but the patterns are actually found using a Gaussian mixture model (GMM), the manifold learning techniques are only used for dimensionality reduction before applying the GMM. In contrast, for example a spectral clustering method would be much closer to "manifold learning for pattern detection", especially because it is closely related to Laplacian Eigenmaps and Diffusion Maps. I do not see a GMM as a manifold learning technique (it does not have a manifold assumption on the data), but the authors may convince me otherwise.

Thank you for the comment. We agree that a GMM is not a manifold learning technique. We aimed to leverage manifold learning as a way to identify a low-dimensional embedding for pattern detection; the aim for the GMM step was to make this embedding and relationship between CBG time-series interpretable. To improve the clarity of the abstract, we have modified the sentence to be more precise and match the introduction text:

“Using manifold learning techniques, we find a low-dimensional embedding that enables the identification of patterns of mobility behavior that align with stay-at-home orders, correlate with socioeconomic factors, cluster geographically, reveal subpopulations that likely migrated out of urban areas, and, importantly, link to COVID-19 case counts.”

A much more important point (compared to the issue of terminology around "manifold learning") is that the authors do not discuss the assumptions underlying the linear and nonlinear manifold learning methods they employ. Instead they seem to have tested multiple methods and ultimately used the ones that "reduced the dimensionality of the data and identified a consistent tubular dense structure in the data" (quote from the supplement section 2). It is reasonable to approach the problem in this way, but with a framework to inform policy decisions in mind, and also for an analysis on this scale, checking the assumptions of the methods is crucial. The authors "found that Laplacian Eigenmaps, Locally Linear Embedding, and Isomaps" (again citing from section 2 in the supplement) worked best among the methods they tried, but these three methods are almost identical in what they assume about the data AND they also work almost identically, especially because the authors seem to be using the same distance metric between points (time series) - here, nearest neighbors with Euclidean distance in the ambient space.

The most crucial assumptions of the manifold learning methods above are (a) the data distribution is uniform (!) on the manifold in the ambient space, and (b) the manifold is compact, i.e. the density does not decay to zero toward any boundary. It is hard to argue that both assumptions are satisfied in this particular scenario. This leads me to believe that the alleged "robustness" of the results over different methods and parameters are (a) because the methods work almost the same and (b) the data itself is

also extremely similar between states (all states considered are in the United States, all states were considered in the same time period, all data collection worked the same way, etc.).

There are remedies and updates to the methods that mitigate both the data density (e.g. using Diffusion Maps instead of Laplacian Eigenmaps) and the data density approaching zero (e.g. by using variable bandwidth kernels, or continuous nearest neighbor kernels, see
* doi: 10.3934/fods.2019001
* doi: 10.1016/j.acha.2015.03.002
). In my opinion, it is crucial to either update to these more recent methods, or discuss in detail why the assumptions for Laplacian Eigenmaps either (a) can be disregarded in this particular case, or (b) are satisfied (which I do not think they are). In particular: what would happen if the data was drawn uniformly over space, i.e. was not clustered toward cities? What would happen if GMM was applied to 14 SVD vectors (the same number as for LE), instead of 60? How do extreme outliers influence the results (e.g. faulty time series)? What happens if only half of the data is available, or twice as much (e.g. discussing convergence/ stability under more/less data)?

We thank the reviewer for this comment and suggestions. We appreciate the questions around the underlying assumptions of Laplacian Eigenmaps and pointing us to recently developed extensions of Diffusion Maps that we were unaware of. At your suggestion, we have tested these other methods including Diffusion Maps with fixed **and** variable bandwidth kernels to investigate the underlying assumptions around data density and data density approaching zero as compared to Laplacian Eigenmaps.

In summary, all of the methods (including the new ones) recover a similar result where CBGs align in the order of mean stay-at-home levels similar to Laplacian Eigenmaps for clusters A,B,C, and D. There are some differences in GMM cluster membership. Fixed bandwidth Diffusion Maps and GMM recovers cluster E similarly to Laplacian Eigenmaps and GMMs. However, the variable bandwidth Diffusion Maps and GMMs can capture similar results for Cluster E, but it depends on the parameter inputs and is more variable than the other methods. In our investigation of these results, it appears that the variable bandwidth Diffusion maps recovers a similar embedding to the other methods, but it becomes more challenging for the GMM to identify cluster E. This may indeed be due to the difference in the underlying assumption around compactness and points to future research directions for us with these methods and data.

To be more specific about the results, here is a visualization of the results across the Laplacian Eigenmaps, fixed bandwidth kernel Diffusion maps, and variable bandwidth kernel Diffusion maps, respectively:

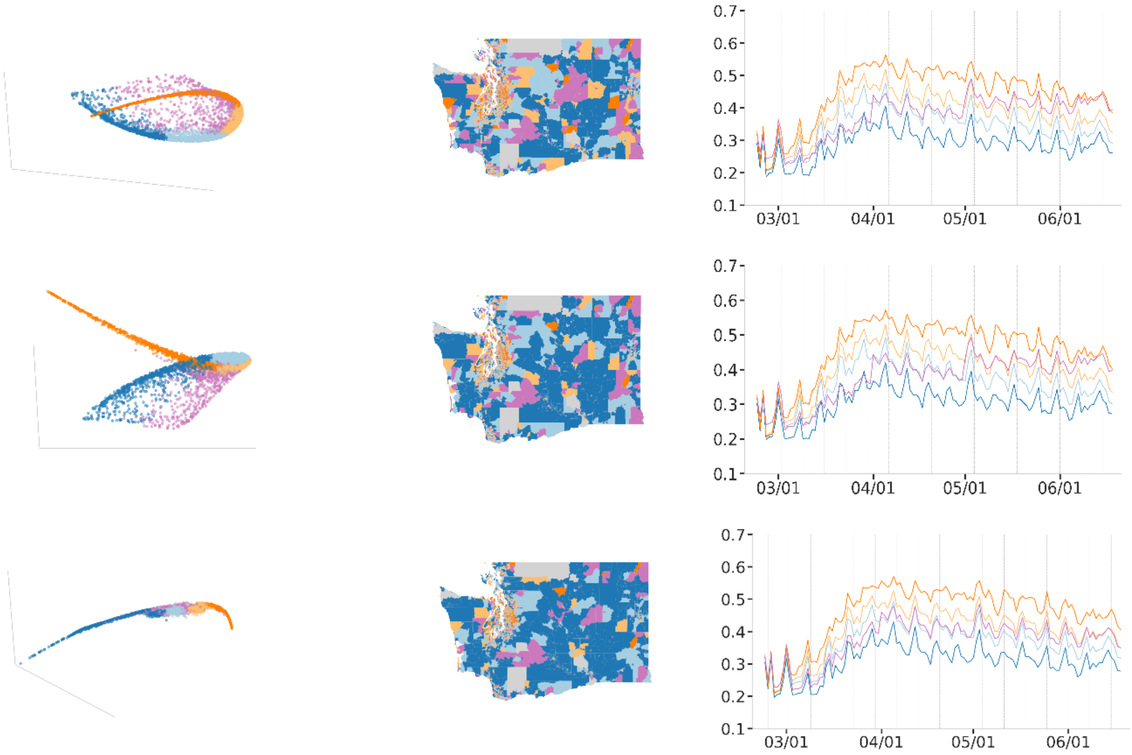

Note that the aggregated time-series clusters look similar across the different variations of Diffusion Maps. As noted earlier, there are differences in cluster membership including cluster E For transparency, we also show how the variable bandwidth Diffusion Map results change as the neighbor parameter k = 100 and 250 in the following figure:

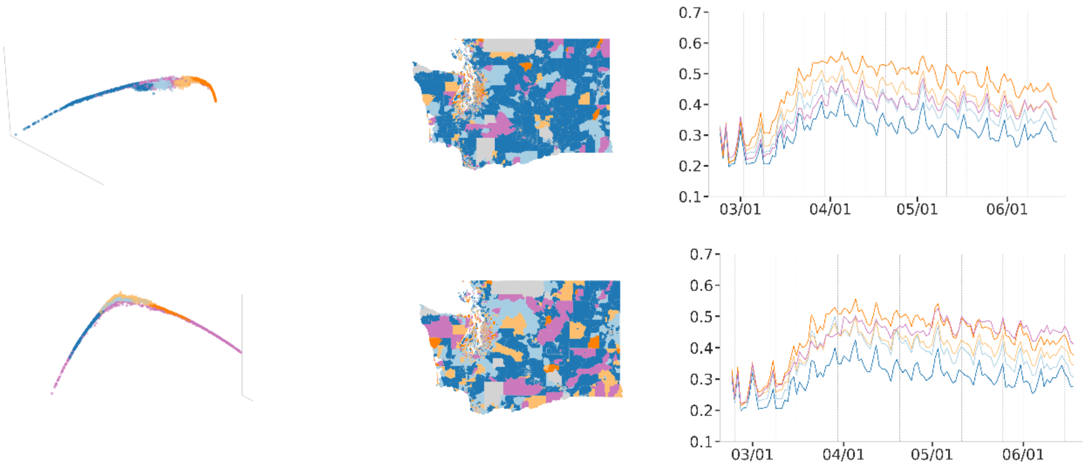

The GMM for the k=250 case recruits more CBGs in cluster E. The spike in the time-series (that is described in the manuscript) is still apparent, but it does raise the overall stay-at-home percentage for cluster E. We have added these results to the supplement (Supplement Figure S3) along with a detailed description about the impact of varying the parameters in Supplement Section 2.

Despite there being some differences in the results of these methods, we believe that there is a significant amount of quantitative similarity and helps validate the method and approach. We thank the reviewer for pointing us to these methodologies and their underlying assumptions on the data distribution. We believe these new analyses and results substantially strengthen the analysis in the paper and the robustness of the results in the main manuscript. We have chosen to keep the Laplacian Eigenmap versions of the figures and results in the main manuscript, but we have made several modifications to the main manuscript and supplement to report these new methodologies and the impact of varying parameters. Specifically, we have added to the methods section, results sections, and discussion. We have also added a supplement section with both text and new figures from these Diffusion Maps results. In addition, we have added citations for these methods in the main manuscript and supplement.

Regarding the statistical tests, I have less severe - but similar - concerns toward discussion of assumptions:

Two main statistical tests are used, Kolmogorov-Smirnov (to test if the speed to respond to stay-at-home orders in March 2020 is the same between identified clusters) and Jonckheere–Terpstra (to test if median stay-at-home fraction, population density, and household income of CBGs had a consistently decreasing trend for four of the five clusters).
I am not a statistician, so I cannot judge whether there are underlying, inherent flaws in using these tests for this particular data, but they seem appropriate for the tasks.
Nevertheless, the assumptions (and if/why they apply) of the two tests could be discussed in more detail, e.g. normality assumption for the individual speed distributions for the Kolmogorov-Smirnov test. For the Jonckheere–Terpstra test, these assumptions may be harder to rationalize, see in particular Assumption 3 (independence of observations) and 4 (the distributions of the scores must be the same with the only possible difference being a shift in location) here:
https://statistics.laerd.com/spss-tutorials/jonckheere-terpstra-test-using-spss-statistics.php#assumptions

We added a sentence to the Methods section that describes the hypotheses compared by the Jonckheere-Terpstra test. We chose it because it is a rank-based test to study trends in medians across categories. You are correct that the Jonckheere-Terpstra test does have an independence assumption, and the mobility clusters meet that criterion. For example, the income of CBGs in the most mobile

cluster should be independent of the income in the least mobile. We did not formally test the shape and distribution of covariates (assumption 4), but we did not anticipate differences or notice any after inspection of histograms of the covariates.

The Jonckheere--Terpstra test's null hypothesis is that covariate values are from the same distribution across clusters and the alternative is that the median covariate values are in an a priori order (i.e., are increasing or decreasing from cluster A to cluster D).

We have also added a sentence to the Methods section that describes the normality assumptions for the KS test.
To test the difference between clusters in the speed at which CBGs increased their stay-at-home behavior, we used the Kolmogorov-Smirnov[55,56] test as implemented in kstest function of scipy.stats package in Python 3; here, we assume these speeds are drawn from a normal distribution.

Related: figure 5 shows bar charts scaled to 100% that include data with N as small as 2 and 3. This may lead people to the wrong conclusions, because e.g. "same house, Georgia" and "same house, Texas" charts make it look like clusters B and C do not occur at the beginning, but this may just be because of missing data.

Thank you for calling attention to this. We added a comment in the caption calling attention to the bins with very small numbers.

On a more subjective note, I feel that the paper will influence thinking in the field of cell phone mobility data analysis and be of interest to a wider computational science community of researchers; in particular for research with access to large volumes of time series data related to human activities (tracking devices for sport, cell phones, driving data from cars, etc).

I still think the work is highly relevant and important, and should eventually be published. The methodology the authors proposed - even with the flaws regarding their assumptions I outlined above - is still relatively recent and definitely an outstanding feature in the area of the analysis of mobility data. From an applied perspective, the authors show different behaviors identified with their methodology for the early months of the epidemic are associated with cases later in the epidemic, which may allow very important findings in the future.

Thank you for the kind comments.

Some technical issues:

-> regarding "Figure legend": Figure 4 describes the error bar in its caption, "Whiskers span the 95% range."

The caption of Figure describes whiskers represent the observed 95% ranges, not error bars.

-> Figure 7 shows standard deviations (?) around mean curves, without clear explanation. What does "inter-quartile range" mean?

We rewrote the caption. The curves are the medians, and the shaded regions show the 25th through 75th percentiles of the distribution of values observed in the CBGs each day.

-> Abstract: "As COVID-19 cases resurge in the United States" will sound strange in 10 years (I hope...)

True! We removed that phrase.

-> What about people that do not use smartphones? In the US that may be not an issue, but maybe in other countries. Do the authors just assume everybody has one, or are all conclusions "for the population of smartphone users"?

This is part of the more general problem of representativeness of the dataset, which we write about in the Discussion. For example, reference [52] shows that the data covers counties with high and low median incomes, but we can't determine coverage at an individual level.

-> 4.3: "these four clusters come from the same distribution of values (p<0.01)" should list the exact p value

We did not give specific p-values from each of the Jonckheere-Terpstra tests because there were 12 tests (4 states and 3 covariates) all were below the value reported in the text.

-> 4.4: "never-near-home" devices is a little strange; how do the authors know where the home of a device is if it is "never" home?

The "home" location is defined by locations in the recent past, so if a phone begins spending more time in a different location for sufficiently long (about 6 weeks) the "home" location is reassigned. We added a new supporting Figure (S17) that illustrates this point using the Oregon wildfires in September 2020. We moved the text describing how SafeGraph assigns "home" to Methods, and the second paragraph in that section of Results describes how devices that have relocated appear to be "never" home.

-> page 17: "affect the persistence of risk for coronavirus susceptibility and transmission" should replace "coronavirus" with SARS-COV-2 (unless the authors mean all types of coronaviruses, which would be strange)

These have been changed.

Decision Letter, first revision:

natureresearch

Dear Dr. Proctor,

Thank you for submitting your revised manuscript "Insights into population behavior during the COVID-19 pandemic from cell phone mobility data and manifold learning" (NATCOMPUTSCI-21-0229A). It has now been seen by the original referees and their comments are below. The reviewers find that the paper has improved in revision, and therefore we'll be happy in principle to publish it in Nature Computational Science, pending minor revisions to satisfy the referees' final requests and to comply with our editorial and formatting guidelines.

We are now performing detailed checks on your paper and will send you a checklist detailing our editorial and formatting requirements in about a week. Please do not upload the final materials and make any revisions until you receive this additional information from us.

TRANSPARENT PEER REVIEW
Nature Computational Science offers a transparent peer review option for new original research manuscripts submitted from 17th February 2021. We encourage increased transparency in peer review by publishing the reviewer comments, author rebuttal letters and editorial decision letters if the authors agree. Such peer review material is made available as a supplementary peer review file. Please state in the cover letter ‘I wish to participate in transparent peer review’ if you want to opt in, or ‘I do not wish to participate in transparent peer review’ if you don’t. Failure to state your preference will result in delays in accepting your manuscript for publication.
Please note: we allow redactions to authors’ rebuttal and reviewer comments in the interest of confidentiality. If you are concerned about the release of confidential data, please let us know specifically what information you would like to have removed. Please note that we cannot incorporate redactions for any other reasons. Reviewer names will be published in the peer review files if the reviewer signed the comments to authors, or if reviewers explicitly agree to release their name. For more information, please refer to our FAQ page.

Thank you again for your interest in Nature Computational Science Please do not hesitate to contact me if you have any questions.

Best,
Fernando

--
Fernando Chirigati, PhD
Chief Editor, Nature Computational Science

Nature Portfolio

ORCID
IMPORTANT: Non-corresponding authors do not have to link their ORCIDs but are encouraged to do so. Please note that it will not be possible to add/modify ORCIDs at proof. Thus, please let your co-authors know that if they wish to have their ORCID added to the paper they must follow the procedure described in the following link prior to acceptance:
https://www.springernature.com/gp/researchers/orcid/orcid-for-nature-research

Reviewer #1 (Remarks to the Author):

The authors have done an admirable amount of work to better present the mobility data. While questions remain I understand that these may be difficult to answer with the data sharing agreement.

Reviewer #3 (Remarks to the Author):

The authors have addressed my concerns and I have no further comments.

Reviewer #4 (Remarks to the Author):

The authors have included a lot of new and important results regarding Diffusion Maps, which significantly add to robustness of their work. The change from general "policy makers" to "local epidemiologists" is also very welcome, and makes the target audience precise.

My concerns regarding assumptions both for LLE and the statistical tests have been addressed appropriately.

The only issue I still have is that the code base seems to not be updated with the new diffusion map implementations, and the paper (and supplement) also does not detail how exactly they were implemented. Updating the code and making a reference to it in the text (which is already there) should be enough.

Author Rebuttal, first revision:

Remarks to the Author:
The authors have done an admirable amount of work to better present the mobility data. While questions remain I understand that these may be difficult to answer with the data sharing agreement.

Fantastic.

Reviewer #3:
Remarks to the Author:
The authors have addressed my concerns and I have no further comments.

Great!

Reviewer #4:
Remarks to the Author:
The authors have included a lot of new and important results regarding Diffusion Maps, which significantly add to robustness of their work. The change from general "policy makers" to "local epidemiologists" is also very welcome, and makes the target audience precise.

Great to hear as well.

My concerns regarding assumptions both for LLE and the statistical tests have been addressed appropriately.

Wonderful.

The only issue I still have is that the code base seems to not be updated with the new diffusion map implementations, and the paper (and supplement) also does not detail how exactly they were implemented. Updating the code and making a reference to it in the text (which is already there) should be enough.

We have made changed to the code repository and modifications to the supplement to address this issue.

Final Decision Letter:

naturerescarch

Dear Dr Proctor,

We are pleased to inform you that your Article "Insights into population behavior during the COVID-19 pandemic from cell phone mobility data and manifold learning" has now been accepted for publication in Nature Computational Science.

In approximately 10 business days you will receive an email with a link to choose the appropriate publishing options for your paper and our Author Services team will be in touch regarding any additional information that may be required.

Please note that *Nature Computational Science* is a Transformative Journal (TJ). Authors may publish their research with us through the traditional subscription access route or make their paper immediately open access through payment of an article-processing charge (APC). Authors will not be required to make a final decision about access to their article until it has been accepted. Find out more about Transformative Journals

Authors may need to take specific actions to achieve compliance with funder and institutional open access mandates. For submissions from January 2021, if your research is supported by a funder that requires immediate open access (e.g. according to Plan S principles) then you should select the gold OA route, and we will direct you to the compliant route where possible. For authors selecting the subscription publication route our standard licensing terms will need to be accepted, including our self-archiving policies. Those standard licensing terms will supersede any other terms that the author or any third party may assert apply to any version of the manuscript.

You will not receive your proofs until the publishing agreement has been received through our system.

If you have any questions about our publishing options, costs, Open Access requirements, or our legal forms, please contact ASJournals@springernature.com

Acceptance of your manuscript is conditional on all authors' agreement with our publication policies (see https://www.nature.com/natcomputsci/for-authors). In particular your manuscript must not be published elsewhere and there must be no announcement of the work to any media outlet until the publication date (the day on which it is uploaded onto our web site).

Before your manuscript is typeset, we will edit the text to ensure it is intelligible to our wide readership and conforms to house style. We look particularly carefully at the titles of all papers to ensure that they are relatively brief and understandable.

Once your manuscript is typeset and you have completed the appropriate grant of rights, you will

receive a link to your electronic proof via email with a request to make any corrections within 48 hours. If, when you receive your proof, you cannot meet this deadline, please inform us at rjsproduction@springernature.com immediately.

If you have queries at any point during the production process then please contact the production team at rjsproduction@springernature.com. Once your paper has been scheduled for online publication, the Nature press office will be in touch to confirm the details.

Content is published online weekly on Mondays and Thursdays, and the embargo is set at 16:00 London time (GMT)/11:00 am US Eastern time (EST) on the day of publication. If you need to know the exact publication date or when the news embargo will be lifted, please contact our press office after you have submitted your proof corrections. Now is the time to inform your Public Relations or Press Office about your paper, as they might be interested in promoting its publication. This will allow them time to prepare an accurate and satisfactory press release. Include your manuscript tracking number NATCOMPUTSCI-21-0229B and the name of the journal, which they will need when they contact our office.

About one week before your paper is published online, we shall be distributing a press release to news organizations worldwide, which may include details of your work. We are happy for your institution or funding agency to prepare its own press release, but it must mention the embargo date and Nature Methods. Our Press Office will contact you closer to the time of publication, but if you or your Press Office have any inquiries in the meantime, please contact press@nature.com.

An online order form for reprints of your paper is available at https://www.nature.com/reprints/author-reprints.html. All co-authors, authors' institutions and authors' funding agencies can order reprints using the form appropriate to their geographical region.

We welcome the submission of potential cover material (including a short caption of around 40 words) related to your manuscript; suggestions should be sent to Nature Computational Science as electronic files (the image should be 300 dpi at 210 x 297 mm in either TIFF or JPEG format). We also welcome suggestions for the Hero Image, which appears at the top of our home page; these should be 72 dpi at 1400 x 400 pixels in JPEG format. Please note that such pictures should be selected more for their aesthetic appeal than for their scientific content, and that colour images work better than black and white or grayscale images. Please do not try to design a cover with the Nature Computational Science logo etc., and please do not submit composites of images related to your work. I am sure you will understand that we cannot make any promise as to whether any of your suggestions might be selected for the cover of the journal.

You can now use a single sign-on for all your accounts, view the status of all your manuscript

submissions and reviews, access usage statistics for your published articles and download a record of your refereeing activity for the Nature journals.

To assist our authors in disseminating their research to the broader community, our SharedIt initiative provides you with a unique shareable link that will allow anyone (with or without a subscription) to read the published article. Recipients of the link with a subscription will also be able to download and print the PDF.

As soon as your article is published, you will receive an automated email with your shareable link.

We look forward to publishing your paper.

Best,
Fernando
